# Fine Mapping Major Histocompatibility Complex Associations in Psoriasis and Its Clinical Subtypes

**DOI:** 10.1016/j.ajhg.2014.07.002

**Published:** 2014-08-07

**Authors:** Yukinori Okada, Buhm Han, Lam C. Tsoi, Philip E. Stuart, Eva Ellinghaus, Trilokraj Tejasvi, Vinod Chandran, Fawnda Pellett, Remy Pollock, Anne M. Bowcock, Gerald G. Krueger, Michael Weichenthal, John J. Voorhees, Proton Rahman, Peter K. Gregersen, Andre Franke, Rajan P. Nair, Gonçalo R. Abecasis, Dafna D. Gladman, James T. Elder, Paul I.W. de Bakker, Soumya Raychaudhuri

**Affiliations:** 1 Department of Human Genetics and Disease Diversity, Graduate School of Medical and Dental Sciences, Tokyo Medical and Dental University, Tokyo 113-0085, Japan; 2 Laboratory for Statistical Analysis, RIKEN Center for Integrative Medical Sciences, Yokohama 230-0045, Japan; 3 Division of Rheumatology, Immunology, and Allergy, Brigham and Women’s Hospital and Harvard Medical School, Boston, MA 02115, USA; 4 Division of Genetics, Brigham and Women’s Hospital and Harvard Medical School, Boston, MA 02115, USA; 5 Program in Medical and Population Genetics, Broad Institute, Cambridge, MA 02142, USA; 6 Department of Biostatistics and Center for Statistical Genetics, University of Michigan, Ann Arbor, MI 48109, USA; 7 Department of Dermatology, University of Michigan Medical School, Ann Arbor, MI 48109, USA; 8 Institute of Clinical Molecular Biology, Kiel University, Kiel 24105, Germany; 9 Division of Rheumatology, Department of Medicine, University of Toronto, Toronto, ON M5T 2S8, Canada; 10 Centre for Prognosis Studies in the Rheumatic Diseases, Toronto Western Research Institute, University of Toronto, Toronto, ON M5T 2S8, Canada; 11 National Heart and Lung Institute, Imperial College, London SW7 2AZ, UK; 12 Department of Dermatology, University of Utah, Salt Lake City, UT 84112, USA; 13 Department of Dermatology, Christian-Albrechts-Universität zu Kiel, Kiel 24105, Germany; 14 Memorial University of Newfoundland, St. John’s, NL A1C5S7, Canada; 15 The Feinstein Institute for Medical Research, North Shore – Long Island Jewish Health System, Manhasset, NY 11030, USA; 16 Toronto Western Research Institute, University of Toronto, Toronto, ON M5G 2M9, Canada; 17 Ann Arbor Veterans Affairs Hospital, Ann Arbor, MI 48105, USA; 18 Department of Medical Genetics, Center for Molecular Medicine, University Medical Center Utrecht, Utrecht 3584 CG, the Netherlands; 19 Department of Epidemiology, Julius Center for Health Sciences and Primary Care, University Medical Center Utrecht, Utrecht 3584 CG, the Netherlands; 20 Arthritis Research UK Epidemiology Unit, Centre for Musculoskeletal Research, Institute of Inflammation and Repair, University of Manchester, Manchester M13 9PT, UK

## Abstract

Psoriasis vulgaris (PsV) risk is strongly associated with variation within the major histocompatibility complex (MHC) region, but its genetic architecture has yet to be fully elucidated. Here, we conducted a large-scale fine-mapping study of PsV risk in the MHC region in 9,247 PsV-affected individuals and 13,589 controls of European descent by imputing class I and II human leukocyte antigen (HLA) genes from SNP genotype data. In addition, we imputed sequence variants for *MICA*, an MHC HLA-like gene that has been associated with PsV, to evaluate association at that locus as well. We observed that *HLA-C^∗^06:02* demonstrated the lowest p value for overall PsV risk (p = 1.7 × 10^−364^). Stepwise analysis revealed multiple *HLA-C^∗^06:02*-independent risk variants in both class I and class II HLA genes for PsV susceptibility (*HLA-C^∗^12:03*, HLA-B amino acid positions 67 and 9, HLA-A amino acid position 95, and HLA-DQα1 amino acid position 53; p < 5.0 × 10^−8^), but no apparent risk conferred by *MICA*. We further evaluated risk of two major clinical subtypes of PsV, psoriatic arthritis (PsA; n = 3,038) and cutaneous psoriasis (PsC; n = 3,098). We found that risk heterogeneity between PsA and PsC might be driven by HLA-B amino acid position 45 (p_omnibus_ = 2.2 × 10^−11^), indicating that different genetic factors underlie the overall risk of PsV and the risk of specific PsV subphenotypes. Our study illustrates the value of high-resolution HLA and *MICA* imputation for fine mapping causal variants in the MHC.

## Introduction

Psoriasis vulgaris (PsV [MIM 177900]) is a common immune-mediated skin disease characterized by epidermal hyperplasia, inflammatory cell infiltration, and vascular remodeling.^1,2^ Approximately one-third of PsV-affected individuals develop a related inflammatory musculoskeletal condition called psoriatic arthritis (PsA), which is considered more severe than the other more common PsV subtype, cutaneous psoriasis (PsC).^3^ Previous linkage and association studies have mapped PsV risk to a critical region spanning ∼300 kb within the major histocompatibility complex (MHC) class I region on 6p21 (this region is termed *PSORS1*).^4^ Subsequent analyses have identified *HLA-Cw6* (MIM 142840) as the risk allele of *PSORS1*.^5,6^ Although recent large-scale genome-wide association studies (GWASs) have identified more than 35 genetic risk loci for PsV outside the MHC region, *HLA-C^∗^06:02* (the most frequent four-digit allele equivalent to *HLA-Cw6*) has consistently demonstrated the strongest association with PsV risk.^7–12^

However, the effects of the genetic architecture of the MHC region on PsV risk have yet to be fully elucidated. Previous studies have suggested the existence of other risk variants in addition to *HLA-C^∗^06:02* in class I human leukocyte antigen (HLA) genes (*HLA-A* [MIM 142800] or *HLA-B* [MIM 142830]) and class II HLA genes (*HLA-DRB1* [MIM 142857], *HLA-DQA1* [MIM 146880], *HLA-DQB1* [MIM 604305], *HLA-DPA1* [MIM 142880], or *HLA-DPB1* [MIM 142858]).^13–18^ Investigators have also studied polymorphisms of MHC class I polypeptide-related sequence A (*MICA* [MIM 600169]), an HLA-like gene that does not present antigen.^17,19,20^ However, strong and complex linkage-disequilibrium (LD) patterns in the MHC region^21,22^ have thus far challenged the identification of independent risk signals. Moreover, analyses focusing on the two major subsets of PsV have identified different effect sizes of associated risk alleles (including *HLA-C^∗^06:02*) between PsA and PsC, suggesting heterogeneous effects of HLA alleles on the two major subphenotypes.^13–16,23^

Recently, we devised an analytical approach to fine map risk of the variants in the MHC region by imputing genotypes of amino acid polymorphisms in the classical HLA genes and classical two- and four-digit alleles.^24–26^ Each classical four-digit HLA allele corresponds to a unique amino acid sequence encoded by the HLA gene, and thus polymorphic residues at each amino acid position could also be targets of disease-risk association studies. This approach has been used for successfully fine mapping HLA alleles of several autoimmune or immune-mediated diseases, including durable host control of HIV infection (MIM 609423), seropositive and seronegative rheumatoid arthritis (MIM 180300), myasthenia gravis (MIM 254200), and follicular lymphoma (MIM 613024).^25–29^ In certain instances, our approach has been able to pinpoint individual amino acid sites that might account for disease risk within HLA molecules.^25–27^

In this study, we aimed to fully characterize the genetic architecture of the MHC region for PsV. Our goals were (1) to define the set of risk alleles for PsV at the four-digit HLA allele and amino acid resolutions, (2) to examine how the role of *MICA* in PsV compares to that of other HLA genes, and (3) to identify a genetic marker that distinguishes the risk of two subtypes, PsA and PsC. To this end, we applied our HLA-variant imputation approach to large-scale PsV GWASs and Immunochip studies comprising 9,247 affected individuals and 13,589 control individuals of European ancestry. We also expanded our approach to impute *MICA* alleles and MICA amino acid polymorphisms by constructing a *MICA* imputation reference panel. With the imputed MHC sequence variations, including classical HLA genes and *MICA*, we fine mapped the MHC associations with overall PsV risk and specifically focused on risk comparisons between the PsA and PsC subphenotypes.

## Material and Methods

### Samples

We used data from 9,247 PsV-affected individuals and 13,589 control individuals obtained from six case-control PsV data sets, including four GWASs (the Collaborative Association Study of Psoriasis [CASP] and the Genizon, Kiel, and PsA GWASs), a targeted deep follow-up study of CASP (the CASP-DFU), and one Immunochip-based data set of 3,723 affected and 7,595 control subjects (the Psoriasis Association Genetics Extension [PAGE] study), for a total of 9,247 affected and 13,589 control individuals (Table S1, available online).^7,9,12^ Genotype data of the studies were generated and stringently quality-control (QC) filtered as described elsewhere,^7,9,12^ and all samples were confirmed to be unrelated individuals of European ancestry according to self-reported ethnicity and results of principal-component (PC) analysis. All participating individuals provided written informed consent and were recruited according to the protocols approved by the institutional review board of each institution.

### Phenotype Classification

All PsV-affected individuals were diagnosed by a dermatologist. Diagnosis of PsA was confirmed by a rheumatologist according to the Classification Criteria for Psoriatic Arthritis.^30^ Individuals who had had PsV for 10 or more years but no signs of PsA were classified as having PsC. Our data set included 3,038 PsA subjects, 3,098 PsC subjects, and 3,111 subjects of unknown PsA or PsC status (Table S1).

### Statistical Analysis

#### HLA Imputation

For each data set, we used SNP2HLA^24^ to extract SNP genotypes located in the MHC region to impute classical two- and four-digit HLA alleles of and amino acid polymorphisms encoded by the eight class I and class II HLA genes (*HLA-A*, *HLA-B*, *HLA-C*, *HLA-DRB1*, *HLA-DQA1*, *HLA-DQB1*, *HLA-DPA1*, and *HLA-DPB1*). We conducted HLA imputation for each data set separately by using HLA and SNP genotypes from the Type 1 Diabetes Genetics Consortium (T1DGC; n = 5,225), which has demonstrated a high imputation accuracy for classical HLA alleles,^24,26,31^ as a reference panel. We obtained information on HLA-gene polymorphisms from the IMGT/HLA Database.^32^ Amino acid sequences encoded by the imputed HLA genes are indicated in Figure S1. For HLA amino acid positions, we indicate the start codon of the mature HLA protein as position 1, and we label the codon 5′ to this site as −1.^24^ SNP2HLA checks concordance of allele strands of the A/T or G/C SNPs between the data set and the reference panel on the basis of allele-frequency comparison.^24^ We applied postimputation QC criteria of MAF > 0.1% for the association analysis.

#### MICA Imputation

To expand our HLA imputation protocol into HLA-like genes, we constructed a reference panel for imputation of *MICA* variants. We obtained classical four-digit *MICA* alleles for the subjects from a subset of the PsA data set (n = 1,046). These samples were not selected in any particular way. We obtained MICA amino acid sequences from the IMGT/HLA Database^32^ and the encoded MICA amino acid polymorphisms of the subjects, as well as the genotypes of *MICA* classical alleles and the genotyped SNPs in the MHC region. Using the constructed *MICA* reference panel and SNP2HLA,^24^ we imputed *MICA* variants for the other data-set collections. Imputed genotypes of the *MICA* alleles and MICA amino acid polymorphisms were extracted and merged into those obtained from HLA imputation mentioned in the previous section. We empirically assessed the accuracy of imputing *MICA* variants by additionally genotyping *MICA* in a subset of the subjects from the PAGE Immunochip data set (n = 104) and comparing concordances of the imputed and genotyped classical *MICA* variants as described elsewhere.^24,26^

### Statistical Framework for Association Analysis

We used the following analyses to test associations between HLA variants and risk of four binary phenotypes: (1) overall analysis of PsV susceptibility (PsV-affected versus control individuals), (2) stratified analysis of PsA susceptibility (PsA-affected versus control individuals), (3) stratified analysis of PsC susceptibility (PsC-affected versus control individuals), and (4) intra-PsV analysis directly comparing PsA to PsC (PsA-affected versus PsC-affected individuals). For each phenotype, we assessed variant risk with a logistic-regression model assuming additive effects of the allele dosages in the log-odds scale and their fixed effects among the data-set collections. We defined HLA variants to include biallelic SNPs in the MHC region, two- and four-digit biallelic classical HLA or *MICA* alleles, biallelic HLA or MICA amino acid polymorphisms for respective residues, and multiallelic HLA or MICA amino acid polymorphisms for respective positions. To account for potential population-based and data-set-specific confounding factors, we included the top ten PCs and an indicator variable for each data set as covariates. For HLA variants with *m* alleles (*m* = 2 for biallelic variants and *m* > 2 for multiallelic variants), we included *m* − 1 alleles, excluding the most frequent allele as a reference, as independent variables in the regression model. This resulted in the following logistic-regression model:

$$\log\left(\text{odds}\right)=\beta_{0}+\sum_{j=1}^{m-1}\beta_{1,j}x_{j}+\sum_{k=1}^{K}\left(\sum_{l=1}^{L}\beta_{2,k,l}y_{k,l}+\beta_{3,k}z_{k}\right)+\varepsilon ,$$

where *β*_*0*_ is the logistic-regression intercept and *β*_*1,j*_ is the additive effect of the dosage of allele *j* for the variant *x*_*j*_. *K* and *L* are numbers of the collections and PCs enrolled in the analysis. *y*_*k,l*_ is the *l*^th^ PC for the *k*^th^ collection, and *z*_*k*_ is the indicator variable for the collection-specific intercept. *β*_*2,k,l*_ and *β*_*3,k*_ parameters are the effects of *y*_*k,l*_ and *z*_*k*_, respectively. An omnibus p value of the variant (p_omnibus_) was obtained by a log-likelihood ratio test comparing the likelihood of the null model against the likelihood of the fitted model. We assessed the significance of the improvement in fit by calculating the deviance (−2 × the log likelihood ratio), which follows a χ^2^ distribution with *m* − 1 degree(s) of freedom.

### Conditional Association Analysis

For conditional association analysis, we considered the regression model including the additional HLA variants as covariates. When conditioning on specific HLA amino acid position(s), we included multiallelic variants of the amino acid residues as covariates. When conditioning on specific HLA gene(s), we included all two- and four-digit classical alleles of the HLA gene(s) (but not alleles with strong correlations [*R*^*2*^ > 0.97]). We consecutively selected the HLA variants to be included as covariates for each HLA gene separately in a forward-type stepwise fashion until no variant satisfied the genome-wide significant threshold (p < 5.0 × 10^−8^). We tested a multivariate full regression model by including the *HLA-C*, *HLA-B*, *HLA-A*, and *HLA-DQA1* risk variants identified by the stepwise regression analysis as covariates and excluding the most frequent allele (or residue) from each locus (or amino acid position) as a reference allele (Table 1). Assuming a PsV prevalence of 2.0%, we estimated phenotypic variance explained by the risk HLA alleles and amino acid polymorphisms on the basis of the effect sizes obtained from the multivariate regression analysis and a liability threshold model.^2^

### Testing for Discordant Effect Sizes on PsA and PsC

We tested whether the effect sizes of *m* classical four-digit alleles of the HLA gene had concordant risks between PsA and PsC, as described elsewhere.^26^ For each of the two compared phenotypes (PsA-affected versus control individuals and PsC-affected versus control individuals), we calculated multivariate odds ratios (ORs) of *m* − 1 alleles by including them as binary independent variables in the regression model, where the most frequent allele was excluded as a reference. Let *β*_PsA,1_, …, *β*_PsA,*m*−1_ and *v*_PsA,1_, …, *v*_PsA,*m*−1_ be the multivariate log ORs and their variances, respectively, in PsA-affected versus control individuals, and let *β*_PsC,1_, …, *β*_PsC,*m*−1_ and *v*_PsC,1_, …, *v*_PsC,*m*−1_ be those in PsC-affected versus control individuals. We evaluated discordance of the effect sizes between the compared phenotypes (p_heterogeneity_) by testing the statistic

$$\sum_{i=1}^{m-1}\frac{{\left(\beta_{\text{PsA},i}-\beta_{\text{PsC},i}\right)}^{2}}{v_{\text{PsA},i}+v_{\text{PsC},i}},$$

which follows a χ^2^ distribution with *m* − 1 degrees of freedom under the null hypothesis of concordant effects.

## Results

### HLA and MICA Imputation

After imputation of HLA and *MICA*, we obtained genotypes for 7,078 SNPs in the MHC region (29.6–33.2 Mb at chromosome 6, UCSC Genome Browser hg18), 105 two-digit HLA or *MICA* alleles, 176 four-digit HLA or *MICA* alleles, and 438 amino acid polymorphisms encoded by HLA or *MICA* genes. Imputation of *MICA* demonstrated high concordance between genotyped and imputed genotypes (88.9% for both two- and four-digit alleles), which was comparable to that reported for imputation of other HLA genes.^24–26^

### *HLA-C^∗^06:02* Has the Strongest Association with PsV Risk

**Figure 1:**
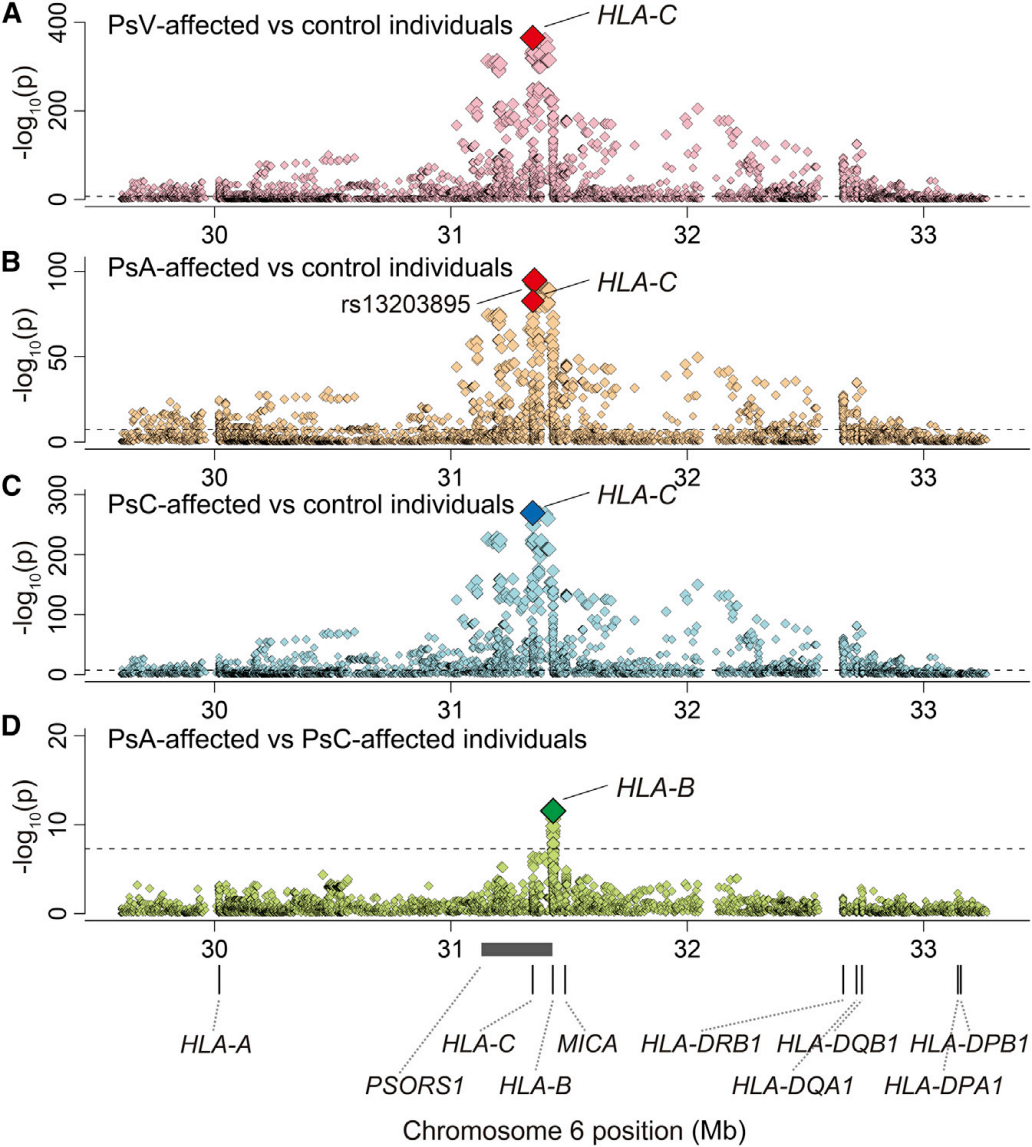
Plots of Nominal Association between the MHC Variants and PsV and Its Subphenotypes of PsA and PsC Each diamond represents the –log_10_(p) of the variants, including SNPs, classical HLA or *MICA* alleles, and amino acid polymorphisms encoded by the HLA genes or *MICA*. The dotted horizontal line represents the significance threshold of p = 5.0 × 10^−8^. The bottom panel shows the physical positions of the HLA genes, *MICA*, and *PSORS1* on chromosome 6 (UCSC Genome Browser hg18). We tested four binary phenotypes: (A) PsV-affected versus control individuals, (B) PsA-affected versus control individuals, (C) PsC-affected versus control individuals, and (D) PsA-affected versus PsC-affected individuals.

**Figure 2:**
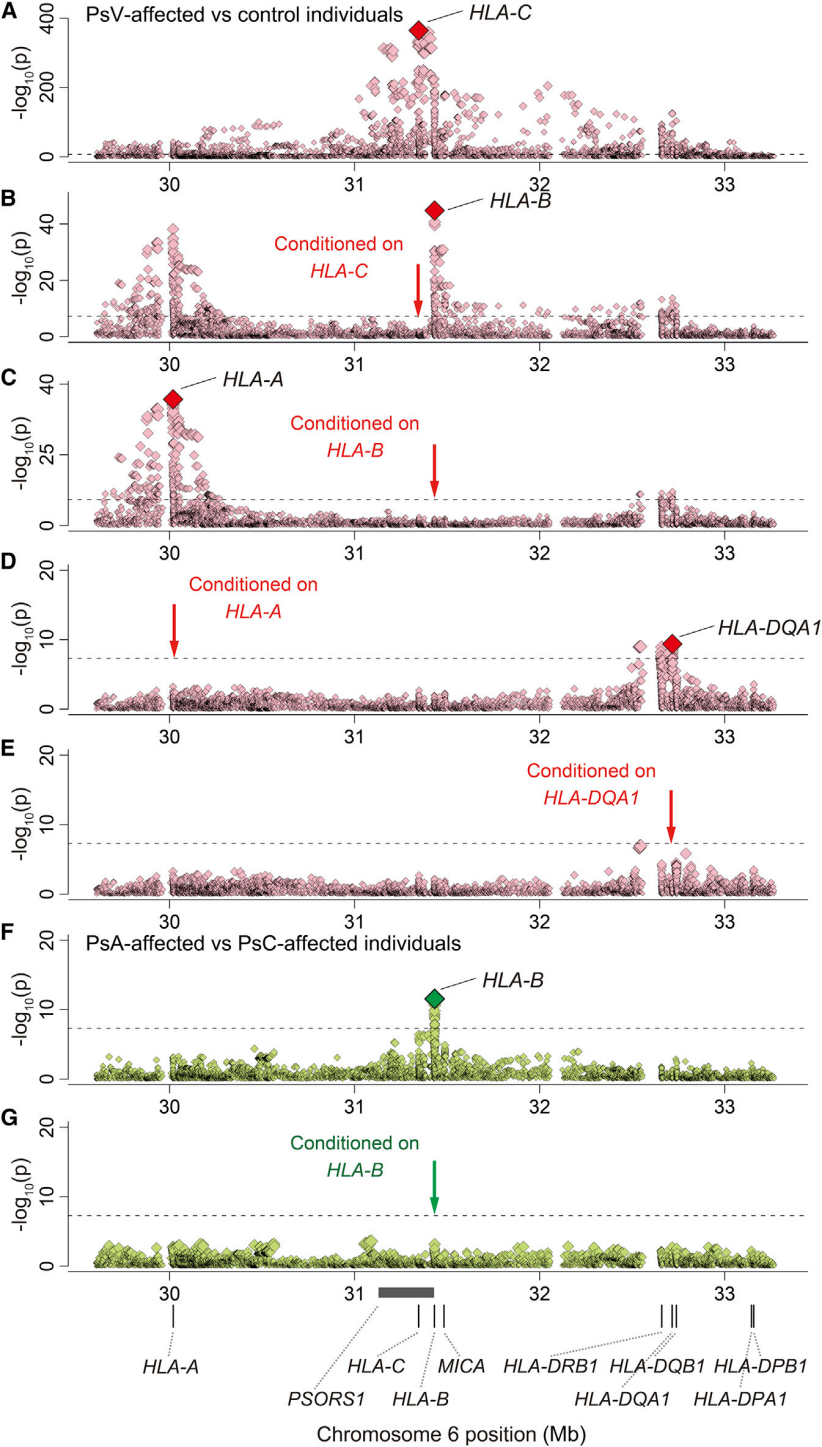
Plots of Stepwise Conditional Association of the Variants in the MHC Region (A–E) Stepwise analysis of *HLA-C*, *HLA-B*, *HLA-A*, and *HLA-DQA1* in PsV-affected versus control individuals. (F and G) Stepwise analysis of *HLA-B* in PsA versus PsC individuals. Each diamond represents the –log_10_(p) of the variants, including SNPs, classical HLA or *MICA* alleles, and amino acid polymorphisms encoded by the HLA genes or *MICA*. The dotted horizontal line represents the significance threshold of p = 5.0 × 10^−8^. The most strongly associated amino acid polymorphisms and HLA classical alleles are labeled when their associations satisfied p < 5.0 × 10^−8^.

**Figure 3:**
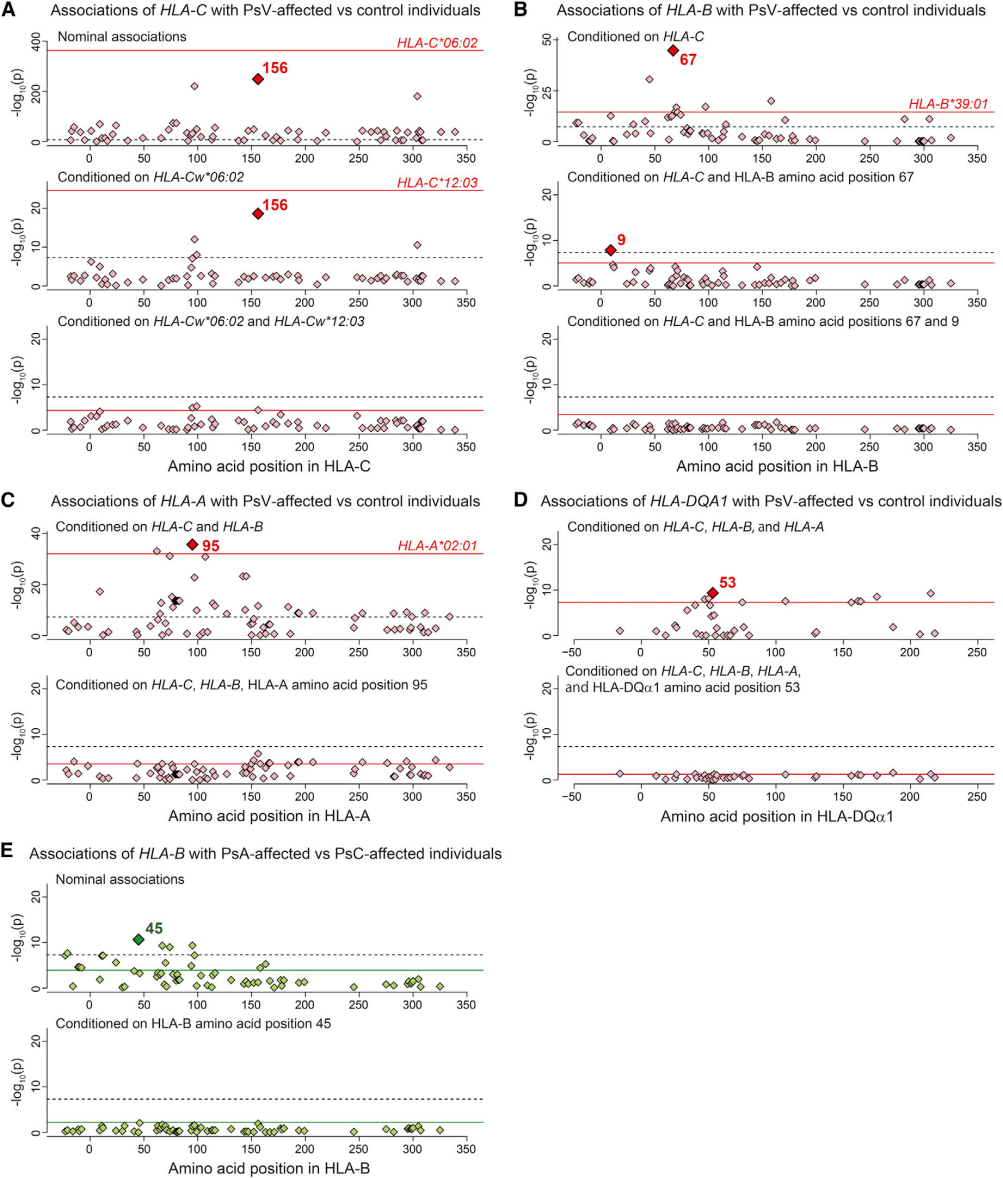
Plots of Stepwise Conditional Association of the HLA Amino Acid Polymorphisms and Classical HLA Alleles (A–D) Stepwise analysis of *HLA-C*, *HLA-B*, *HLA-A*, and *HLA-DQA1* in PsV-affected versus control individuals. (E) Stepwise analysis of *HLA-B* in PsA-affected versus PsC-affected individuals. Each diamond represents the –log_10_(p_omnibus_) of the amino acid polymorphism encoded by the HLA gene. The colored horizontal line represents the –log_10_(p_omnibus_) of the most strongly associated classical allele of the HLA gene. The dotted horizontal line represents the significance threshold of p = 5.0 × 10^−8^. The most strongly associated amino acid polymorphisms and HLA classical alleles are labeled when their associations satisfied p < 5.0 × 10^−8^.

Unsurprisingly, when we tested the imputed variants in the MHC region for overall PsV risk (PsV-affected versus control individuals), the top association signal mapped to *HLA-C* (Figures 1A and 2A; Table S2). The most strongly associated variant was the classical *HLA-C^∗^06:02* allele (p = 1.7 × 10^−364^), highly consistent with previous reports that *HLA-C^∗^06:02* has the strongest association with PsV risk.^7–12,17,18^ We observed that no HLA-C amino acid polymorphism was more strongly associated than *HLA-C^∗^06:02* (the smallest p value, p_omnibus_ = 4.5 × 10^−250^, was at HLA-C amino acid position 156; Figure 3A). When conditioning on *HLA-C^∗^06:02*, we observed the top association signal at the four-digit classical allele of *HLA-C^∗^12:03* (p = 2.5 × 10^−25^). After conditioning on *HLA-C^∗^06:02* and *HLA-C^∗^12:03*, we found that no association exceeded the genome-wide significance threshold for the *HLA-C* variants (p > 5.0 × 10^−8^). These results suggest that multiple classical *HLA-C* alleles, exemplified by *HLA-C^∗^06:02*, explain the influence of *HLA-C* on PsV risk.

### PsV Risk Is Associated with Multiple Class I and Class II HLA Genes

We then investigated additional HLA-variant PsV risk independent of *HLA-C*. When we conditioned on all classical *HLA-C* alleles, we observed a significant independent association at HLA-B amino acid position 67 (p_omnibus_ = 1.8 × 10^−45^; Figures 2B and 3B). Stepwise regression analysis of *HLA-B* variants further identified an independent association at position 9 (p_omnibus_ = 1.5 × 10^−8^). When we conditioned on *HLA-C* and *HLA-B*, we observed a significant independent association at HLA-A amino acid position 95 (p_omnibus_ = 2.1 × 10^−36^; Figures 2C and 3C). Stepwise analysis did not identify additional association within *HLA-A*. When we conditioned on the effects of *HLA-C*, *HLA-B*, and *HLA-A*, we observed a significant independent association at HLA-DQα1 amino acid position 53 (p_omnibus_ = 4.2 × 10^−10^; Figures 2D and 3D). Stepwise analysis did not identify an additional association within *HLA-DQA1*. When conditioning on *HLA-C*, *HLA-B*, *HLA-A*, and *HLA-DQA1*, we observed no other significant associations (p > 5.0 × 10^−8^; Figure 2E). These results demonstrate that PsV risk within the MHC region can be explained by combinations of multiple class I and class II HLA genes.

To highlight specific HLA alleles or amino acid residues that confer overall PsV risk, we defined a multivariate full regression model including all the identified risk variants of *HLA-C*, *HLA-B*, *HLA-A*, and *HLA-DQA1* (Table 1; Table S3). For *HLA-C*, we observed increased risk associated with *HLA-C^∗^06:02* (OR = 3.26, 95% confidence interval [CI] = 3.02–3.52, p = 2.1 × 10^−201^) and *HLA-C^∗^12:03* (OR = 1.38, 95% CI = 1.26–1.52, p = 6.5 × 10^−12^). For *HLA-B*, we observed increased risk associated with Cys67 (OR = 1.56, 95% CI = 1.45–1.67, p = 6.0 × 10^−35^), Met67 (OR = 1.44, 95% CI = 1.30–1.58, p = 2.6 × 10^−13^), and Asp9 (OR = 1.33, 95% CI = 1.21–1.45, p = 1.6 × 10^−9^). HLA-A Val95 (OR = 1.31, 95% CI = 1.25–1.38, p = 4.7 × 10^−28^) and HLA-DQα1 Arg53 (OR = 1.07, 95% CI = 1.01–1.13, p = 0.016) also demonstrated increased risk. In combination, these risk variants explained 6.7% of phenotype variance of PsV under the assumption of 2.0% of disease prevalence,^2^ whereas *HLA-C^∗^06:02* alone explained only 4.9% of the variance.

### Risk Heterogeneity between PsA and PsC Is Explained by *HLA-B*

Next, we conducted an analysis focusing on subphenotypes of PsA and PsC. Overall, the association results of the PsA case-control analysis and of the PsC case-control analysis were similar to those of the PsV case-control analysis (Figures 1A–1C). The classical *HLA-C^∗^06:02* allele demonstrated the lowest p value among the HLA variants in the MHC region. Stepwise association analyses, separately conducted for the PsA case-control analysis and the PsC case-control analysis, both revealed independent contributions of other class I HLA genes (*HLA-B* and *HLA-C*; Figure S2; Table S2). We did not observe independent signals for HLA class II genes in this stratified analysis, perhaps as a result of reduced statistical power.

**Figure 4:**
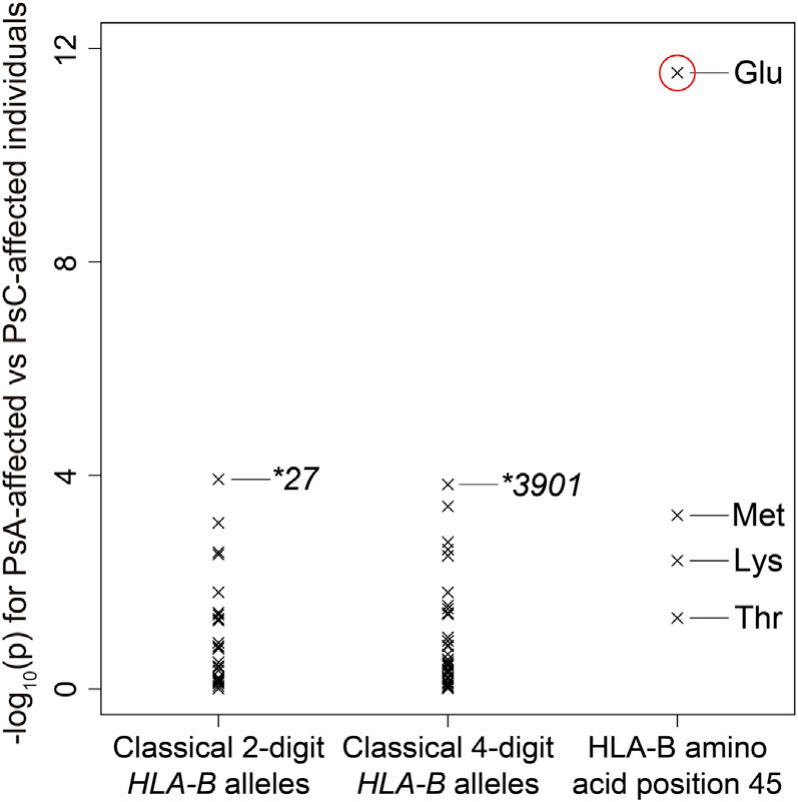
Associations of Classical *HLA-B* Alleles and HLA-B Amino Acid Position 45 for PsA versus PsC Individuals The –log_10_(p) of *HLA-B* classical two- and four-digit *HLA-B* alleles and HLA-B amino acid residues at position 45 in PsA versus PsC individuals. HLA-B Glu45 demonstrated the strongest association (p = 2.9 × 10^−12^; highlighted with a red circle), whereas the *HLA-B* classical alleles showed much smaller effects (p > 1.0 × 10^−4^).

Surprisingly, when we directly assessed comparative risk between PsA and PsC subjects, we found the lowest p value of the nominal association signal at HLA-B amino acid position 45 (p_omnibus_ = 2.2 × 10^−11^; Figures 1D and 2F) rather than at *HLA-C* alleles. After conditioning on HLA-B amino acid position 45, or all classical *HLA-B* alleles, we observed no significant association in the MHC region (p > 5.0 × 10^−8^; Figures 2G and 3E). Of the HLA-B amino acid residues at position 45, HLA-B Glu45 increased PsA susceptibility in comparison to PsC susceptibility (OR = 1.46, 95% CI = 1.31–1.62, p = 2.9 × 10^−12^; Table 2; Table S2). Examining the classical alleles, we noted that *HLA-B^∗^27*, a risk allele for another arthritic disease, ankylosing spondylitis (MIM 106300),^33^ demonstrated the lowest p value for PsA versus PsC association (p = 1.2 × 10^−4^; Figure 4; Table S4) but much less significantly than HLA-B Glu45. We note that *HLA-B^∗^27*, along with *HLA-B^∗^38*, *HLA-B*∗*39*, and a number of other alleles, carries Glu at position 45.

Previous studies comparing PsA and PsC have suggested that *HLA-C^∗^06:02* shows increased PsC risk but decreased PsA risk,^13,14^ and our study replicated this finding in a concordant directional effect (p = 9.4 × 10^−6^ for PsA versus PsC individuals). However, the differential impact of *HLA-C^∗^06:02* on PsA and PsC risk disappeared after we conditioned on HLA-B amino acid position 45 (p = 0.12), suggesting that this reduced effect was the result of linkage to *HLA-B*. In contrast, the effect of HLA-B amino acid position 45 with respect to PsA versus PsC risk retained significance even after we conditioned on *HLA-C^∗^06:02* (p_omnibus_ = 2.2 × 10^−7^).

We evaluated effect-size (OR) heterogeneity in classical four-digit alleles of the HLA genes between the two association analyses of PsA-affected versus control individuals and PsC-affected versus control individuals. Among the eight class I and class II HLA genes that we evaluated in the overall PsV case-control analysis, the *HLA-B* and *HLA-C* alleles showed significant risk heterogeneity (p_heterogeneity_ = 5.8 × 10^−14^ and p_heterogeneity_ = 2.9 × 10^−6^, respectively, with a significance threshold of p < 0.05/8 = 0.0073). When we conditioned on HLA-B amino acid position 45, risk heterogeneity diminished in both *HLA-B* and *HLA-C* classical alleles (p_heterogeneity_ > 0.01). In contrast, when we conditioned on *HLA-C^∗^06:02*, risk heterogeneity diminished for the classical *HLA-C* alleles (p_heterogeneity_ = 0.018), but not in the classical *HLA-B* alleles (p_heterogeneity_ = 6.5 × 10^−5^). These results demonstrate that the risk heterogeneity between PsA and PsC primarily derives from *HLA-B*, but not *HLA-C* (or other), genes.

### No Apparent Contribution of MICA to Psoriasis Risk

*MICA* variants demonstrated nominally significant associations with PsV, PsA, and PsC risk (the lowest p value, p_omnibus_ = 2.2 × 10^−180^, was at MICA amino acid position 298 for PsV-affected versus control individuals; Table S2). However, after we conditioned on *HLA-C* and *HLA-B*, no *MICA* variants showed independent association signals (p > 5.0 × 10^−8^ for any of the four tested phenotype comparisons). Previous studies have suggested independent effects of several *MICA* variants, such as *MICA^∗^016*, *MICA^∗^008:01*, and MICA amino acid position 129, on PsV or its subphenotypes,^19,20^ but our study did not observe apparent associations of these variants when we conditioned on *HLA-C^∗^06:02* or HLA-B amino acid position 67 (p > 0.017). We note that no significant association was observed in the *MICA* variants in direct comparison between PsA subjects and PsC subjects (p > 5.0 × 10^−8^). Therefore, our study could not identify an independent contribution of *MICA* on risk of psoriasis and its clinical subtypes.

## Discussion

In this study, we fine mapped PsV risk within the MHC region. In addition to imputing variants of HLA genes, we evaluated risk of the HLA-like gene *MICA* by creating a *MICA* reference panel that empirically demonstrated high imputation accuracy. Our study identified multiple *HLA-C^∗^06:02*-independent risk variants of both class I and class II HLA genes for PsV susceptibility (*HLA-B*, *HLA-A*, and *HLA-DQA1*), but no apparent risk attributable to *MICA*. We also observed that risk heterogeneity between PsA and PsC could be explained by polymorphisms of a single amino acid site encoded by *HLA-B*, suggesting that different genetic architectures underlie the overall risk of PsV and that of its subphenotypes. To our knowledge, ours is the largest HLA fine-mapping study of PsV associations in the MHC to date and defines genetic heterogeneity between PsA and PsC subphenotypes.

*HLA-C^∗^06:02* has the strongest association with PsV risk, as reported previously.^7–12,17,18^ We demonstrated that no single HLA-C amino acid polymorphism was more strongly associated than the *HLA-C^∗^06:02* classical allele, suggesting that the haplotype sequence including *HLA-C^∗^06:02* itself should be the origin of PsV risk. Several hypotheses might explain this. Clop et al. reported noncoding regulatory variants that are located in enhancer motifs and that are unique to the *HLA-C^∗^06:02* haplotype.^34^ A combination of the polymorphisms in multiple HLA-C amino acid sites could effectively tag *HLA-C^∗^06:02*.^35^ We did observe a more modest independent effect at *HLA-C^∗^12:03* in addition to the large *HLA-C^∗^06:02* effect; Helms et al. reported that *HLA-C^∗^06:02* and *HLA-C^∗^12:03* share several functional domains and peptide-binding pockets of HLA-C.^5^ We note that *HLA-C^∗^12:03* did not show an independent association signal after we conditioned on *HLA-C^∗^06:02* and every classical *HLA-B* allele (p = 0.12), suggesting the possibility that the observed *HLA-C^∗^12:03* association might reflect risk at other *HLA-B* alleles in LD. Further functional studies will be necessary for elucidating the role of *HLA-C* in PsV risk.

**Figure 5:**
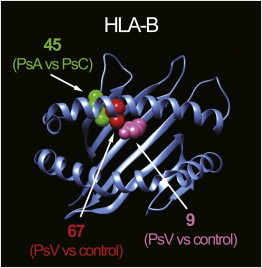
3D Ribbon Models for HLA-B HLA-B structures are based on Protein Data Bank entry 2bvp^36^ and were prepared with UCSF Chimera version 1.7.^37^ Amino acid position 67 and 9 residues associated with overall PsV risk are highlighted as red and pink spheres, respectively. An amino acid position 45 residue associated with subphenotype risk heterogeneity (PsA versus PsC) is highlighted as a green sphere.

In contrast, outside of *HLA-C*, amino acid polymorphisms (HLA-B amino acid positions 67 and 9, HLA-A position 95, and HLA-DQα1 position 53) demonstrated stronger associations than did classical alleles *HLA-B*, *HLA-A*, and *HLA-DQA1*. All of these amino acid sites were located within the HLA antigen binding (Figure 5 and Figure S3). Positions 67 and 9 in HLA-B have been identified in HLA fine-mapping studies for other immune-related diseases.^25–27^ We note that LD structures between the amino acid positions could yield potential ambiguity in fine mapping of the causal amino acid position, and this might be clarified with larger studies.

The contribution of HLA-like genes to immune-related disease risk has long been a topic of discussion.^38,39^ Although our imputation of *MICA* alleles was highly accurate, our study did not observe independent *MICA* risk of PsV after we conditioned on the neighboring risk HLA genes *HLA-C* and *HLA-B*. Previous studies focusing on *MICA* risk did not apply robust conditioning on all classical *HLA-C* and *HLA-B* alleles and thus could have potentially reflected the associations of *HLA-C* and *HLA-B* via LD with them.^19,20^

Here, we were able to successfully decompose the genetic architecture of PsA and PsC to a shared component and a subphenotype-specific component. Our study demonstrates that the HLA gene associated with the risk heterogeneity between PsA and PsC (*HLA-B*) is distinct from the HLA gene most associated with overall PsV risk (*HLA-C*). Previous studies have reported that *HLA-C^∗^06:02* has different effect sizes for PsA and PsC, naturally leading to a hypothesis that the heterogeneity is driven by the difference in *HLA-C*, the major risk factor.^13–16,23^ However, our observation is more concordant with a model where the two PsV subtypes generally share the same risk alleles, including *HLA-C^∗^06:02*, but differ at a specific locus that contributes to subtype differences.

HLA-B amino acid position 45 is the driving MHC position that modulates differential risk of PsA and PsV. The effect of Glu at HLA-B position 45 confers substantial risk of psoriatic arthritis and explains previously reported associations at *HLA-B^∗^27*. This site is located within the binding groove of HLA-B and is classified as one of the functional pockets influencing receptor cell-surface expression or antigen peptide binding or presentation.^40^ Being able to clinically distinguish those individuals who have isolated skin disease (PsC) from those who develop joint disease (PsA) has clinical importance. PsA often occurs in addition to psoriatic skin disease and almost always requires systemic therapy, for example, with anti-TNF or other biologic medications, for the prevention of destructive joint disease. In contrast, PsC can in many instances be managed with topical treatments alone.^3^ Our findings might contribute to utilizing information on HLA variants to improve diagnostic approaches for clinical subphenotypes, as suggested for other complex diseases.^26^ There is a possibility that the HLA-B45 amino acid residue tags other HLA-B driving risk variants at other amino acid sites, although we observed only limited LD between these sites (Figure S4). We also note that our method of evaluating OR heterogeneity (i.e., p_heterogeneity_) might be conservative because of shared control subjects in the PsA and PsC case-control analyses. Further studies will be required for elucidating the functional mechanisms that result in differential PsA and PsC risk.

In summary, our study fine mapped risk of multiple class I and class II HLA genes in PsV and its subphenotypes through large-scale HLA and MICA imputation. Our study should contribute to our understanding of HLA variants in the etiology of PsV.

## Figures and Tables

**Table 1:** Association Results of the HLA Variants for Overall PsV Risk in Multivariate Regression Model

**HLA Variant**	**Frequency**		**PsV-Affected versus Control Individuals**	
	**PsV**	**Control**	**OR (95% CI)**^a^	**p**^a^
**Classical *HLA-C* Alleles**				

*HLA-C^∗^06:02*	0.23	0.093	3.26 (3.02–3.52)	2.1 × 10^−201^
*HLA-C^∗^12:03*	0.073	0.056	1.38 (1.26–1.52)	6.5 × 10^−12^
Other four-digit *HLA-C* alleles	0.70	0.85	reference	reference

**HLA-B Amino Acid Position 67**				

Cys	0.16	0.12	1.56 (1.45–1.67)	6.0 × 10^−35^
Met	0.12	0.046	1.44 (1.30–1.58)	2.6 × 10^−13^
Tyr	0.12	0.16	1.00 (0.93–1.07)	0.93
Phe	0.21	0.26	1.00 (0.93–1.08)	0.99
Ser	0.39	0.42	reference	reference

**HLA-B Amino Acid Position 9**				

Asp	0.096	0.11	1.33 (1.21–1.45)	1.6 × 10^−9^
Tyr	0.70	0.67	reference	reference
His	0.20	0.22	0.87 (0.82–0.92)	1.6 × 10^−6^

**HLA-A Amino Acid Position 95**				

Val	0.34	0.29	1.31 (1.25–1.38)	4.7 × 10^−28^
Ile	0.56	0.59	reference	reference
Leu	0.099	0.12	0.89 (0.83–0.95)	7.0 × 10^−4^

**HLA-DQα1 Amino Acid Position 53**				

Arg	0.37	0.29	1.07 (1.01–1.13)	0.016
Lys	0.39	0.43	reference	reference
Gln	0.25	0.29	0.91 (0.86–0.96)	9.3 × 10^−4^

Abbreviations are as follows: CI, confidence interval; OR, odds ratio; and PsV, psoriasis vulgaris.

a Obtained from the multivariate full regression model including the *HLA-C*, *HLA-B*, *HLA-A*, and *HLA-DQA1* risk variants identified by the stepwise regression analysis.

**Table 2:** Association Results of HLA Variants for Subphenotype Risk Comparisons of PsA and PsC

**HLA Variant**	**Frequency**		**PsA-Affected versus PsC-Affected Individuals**	
	**PsA**	**PsC**	**OR (95% CI)**	**p**
**HLA-B Amino Acid Position 45 (Nominal)**				

Glu	0.43	0.33	1.46 (1.31–1.62)	2.9 × 10^−12^
Thr, Lys, Met	0.57	0.67	reference	reference

Abbreviations are as follows: CI, confidence interval; OR, odds ratio; PsA, psoriatic arthritis; and PsC, cutaneous psoriasis.

## Supplemental Data

Document S1. Figures S1–S4 and Tables S1, S3, and S4

Table S2. Detailed Stepwise Association Results of the HLA Variants for Psoriasis Risk

Document S2. Article plus Supplemental Data

## Web Resources

The URLs for data presented herein are as follows:

IMGT/HLA Database, http://www.ebi.ac.uk/ipd/imgt/hla/
Online Mendelian Inheritance in Man (OMIM), http://omim.org/
RefSeq, http://www.ncbi.nlm.nih.gov/refseq/
SNP2HLA, http://www.broadinstitute.org/mpg/snp2hla/
Type 1 Diabetes Genetics Consortium (T1DGC), https://www.t1dgc.org/home.cfm
UCSC Genome Browser, http://genome.ucsc.edu
