# Supplementary material for: Fine Mapping Major Histocompatibility Complex Associations in Psoriasis and Its Clinical Subtypes
Source: Am J Hum Genet. 2014 Aug 7;95(2):162–72. doi: 10.1016/j.ajhg.2014.07.002 (PMC4129407; doi:10.1016/j.ajhg.2014.07.002)
Supplement: Document S1. Figures S1–S4 and Tables S1, S3, and S4 [file mmc1.pdf]

The American Journal of Human Genetics, Volume *95*

Supplemental Data

Fine Mapping Major Histocompatibility Complex Associations in Psoriasis and Its Clinical Subtypes

Yukinori Okada, Buhm Han, Lam C. Tsoi, Philip E. Stuart, Eva Ellinghaus, Trilokraj Tejasvi, Vinod Chandran, Fawnda Pellett, Remy Pollock, Anne M. Bowcock, Gerald G. Krueger, Michael Weichenthal, John J. Voorhees, Proton Rahman, Peter K. Gregersen, Andre Franke, Rajan P. Nair, Gonçalo R. Abecasis, Dafna D. Gladman, James T. Elder, Paul I.W. de Bakker, and Soumya Raychaudhuri

Figure S1. Amino acid sequences of HLA genes and *MICA*.

HLA alleles*	HLA amino acid positions and residues																																										
	-40	-39	-38	-37	-36	-35	-34	-33	-32	-31	-30	-29	-28	-27	-26	-25	-24	-23	-22	-21	-20	-19	-18	-17	-16	-15	-14	-13	-12	-11	-10	-9	-8	-7	-6	-5	-4	-3	-2	-1	1	2	3	4
*HLA-A*02:01*	x	x	x	x	x	x	x	x	x	x	x	x	x	x	x	x	M	A	V	M	A	P	R	T	L	V	L	L	L	S	G	A	L	A	L	T	Q	T	W	A	G	S	H	S
*HLA-B*07:02*	x	x	x	x	x	x	x	x	x	x	x	x	x	x	x	x	M	L	V	M	A	P	R	T	V	L	L	L	L	S	A	A	L	A	L	T	E	T	W	A	G	S	H	S
*HLA-C*07:01*	x	x	x	x	x	x	x	x	x	x	x	x	x	x	x	x	M	R	V	M	A	P	R	A	L	L	L	L	L	S	G	G	L	A	L	T	E	T	W	A	C	S	H	S
*HLA-DRB1*07:01*	x	x	x	x	x	x	x	x	x	x	x	M	V	C	L	K	L	P	G	G	S	C	M	A	A	L	T	V	T	L	M	V	L	S	S	P	L	A	L	A	G	D	T	Q
*HLA-DQA1*05:01*	x	x	x	x	x	x	x	x	x	x	x	x	x	x	x	x	x	M	I	L	N	K	A	L	M	L	G	A	L	A	L	T	T	V	M	S	P	C	G	G	E	D	I	V
*HLA-DQB1*03:01*	x	x	x	x	x	x	x	x	M	S	W	K	K	A	L	R	I	P	G	G	L	R	A	A	T	V	T	L	M	L	A	M	L	S	T	P	V	A	E	G	R	D	S	P
*HLA-DPA1*01:03*	x	x	x	x	x	x	x	x	x	M	R	P	E	D	R	M	F	H	I	R	A	V	I	L	R	A	L	S	L	A	F	L	L	S	L	R	G	A	G	A	I	K	A	D
*HLA-DPB1*04:01*	x	x	x	x	x	x	x	x	x	x	x	M	M	V	L	Q	V	S	A	A	P	R	T	V	A	L	T	A	L	L	M	V	L	L	T	S	V	V	Q	G	R	A	T	P
*MICA*008:01*	x	x	x	x	x	x	x	x	x	x	x	x	x	x	x	x	x	M	G	L	G	P	V	F	L	L	L	A	G	I	F	P	F	A	P	P	G	A	A	A	E	P	H	S

HLA alleles*	HLA amino acid positions and residues																																										
	5	6	7	8	9	10	11	12	13	14	15	16	17	18	19	20	21	22	23	24	25	26	27	28	29	30	31	32	33	34	35	36	37	38	39	40	41	42	43	44	45	46	47	48
*HLA-A*02:01*	M	R	Y	F	F	T	S	V	S	R	P	G	R	G	E	P	R	F	I	A	V	G	Y	V	D	D	T	Q	F	V	R	F	D	S	D	A	A	S	Q	R	M	E	P	R
*HLA-B*07:02*	M	R	Y	F	Y	T	S	V	S	R	P	G	R	G	E	P	R	F	I	S	V	G	Y	V	D	D	T	Q	F	V	R	F	D	S	D	A	A	S	P	R	E	E	P	R
*HLA-C*07:01*	M	R	Y	F	D	T	A	V	S	R	P	G	R	G	E	P	R	F	I	S	V	G	Y	V	D	D	T	Q	F	V	R	F	D	S	D	A	A	S	P	R	G	E	P	R
*HLA-DRB1*07:01*	P	R	F	L	W	Q	G	K	Y	K	C	H	F	F	N	G	T	E	R	V	Q	F	L	E	R	L	F	Y	N	Q	E	E	F	V	R	F	D	S	D	V	G	E	Y	R
*HLA-DQA1*05:01*	A	D	H	V	A	S	Y	G	V	N	L	Y	Q	S	Y	G	P	S	G	Q	Y	T	H	E	F	D	G	D	E	Q	F	Y	V	D	L	G	R	K	E	T	V	W	C	L
*HLA-DQB1*03:01*	E	D	F	V	Y	Q	F	K	A	M	C	Y	F	T	N	G	T	E	R	V	R	Y	V	T	R	Y	I	Y	N	R	E	E	Y	A	R	F	D	S	D	V	E	V	Y	R
*HLA-DPA1*01:03*	H	V	S	T	Y	A	A	F	V	Q	T	H	R	P	T	G	E	F	M	F	E	F	D	E	D	E	M	F	Y	V	D	L	D	K	K	E	T	V	W	H	L	E	E	F
*HLA-DPB1*04:01*	E	N	Y	L	F	Q	G	R	Q	E	C	Y	A	F	N	G	T	Q	R	F	L	E	R	Y	I	Y	N	R	E	E	F	A	R	F	D	S	D	V	G	E	F	R	A	V
*MICA*008:01*	L	R	Y	N	L	T	V	L	S	W	D	G	S	V	Q	S	G	F	L	A	E	V	H	L	D	G	Q	P	F	L	R	Y	D	R	Q	K	C	R	A	K	P	Q	G	Q

HLA alleles*	HLA amino acid positions and residues																																										
	49	50	51	52	53	54	55	56	57	58	59	60	61	62	63	64	65	66	67	68	69	70	71	72	73	74	75	76	77	78	79	80	81	82	83	84	85	86	87	88	89	90	91	92
*HLA-A*02:01*	A	P	W	I	E	Q	E	G	P	E	Y	W	D	G	E	T	R	K	V	K	A	H	S	Q	T	H	R	V	D	L	G	T	L	R	G	Y	Y	N	Q	S	E	A	G	S
*HLA-B*07:02*	A	P	W	I	E	Q	E	G	P	E	Y	W	D	R	N	T	Q	I	Y	K	A	Q	A	Q	T	D	R	E	S	L	R	N	L	R	G	Y	Y	N	Q	S	E	A	G	S
*HLA-C*07:01*	A	P	W	V	E	Q	E	G	P	E	Y	W	D	R	E	T	Q	N	Y	K	R	Q	A	Q	A	D	R	V	S	L	R	N	L	R	G	Y	Y	N	Q	S	E	D	G	S
*HLA-DRB1*07:01*	A	V	T	E	L	G	R	P	V	A	E	S	W	N	S	Q	K	D	I	L	E	D	R	R	G	Q	V	D	T	V	C	R	H	N	Y	G	V	G	E	S	F	T	V	Q
*HLA-DQA1*05:01*	P	V	L	R	Q	F	R	x	F	D	P	Q	F	A	L	T	N	I	A	V	L	K	H	N	L	N	S	L	I	K	R	S	N	S	T	A	A	T	N	E	V	P	E	V
*HLA-DQB1*03:01*	A	V	T	P	L	G	P	P	D	A	E	Y	W	N	S	Q	K	E	V	L	E	R	T	R	A	E	L	D	T	V	C	R	H	N	Y	Q	L	E	L	R	T	T	L	Q
*HLA-DPA1*01:03*	G	Q	A	F	S	F	E	A	Q	G	G	L	A	N	I	A	I	L	N	N	N	L	N	T	L	I	Q	R	S	N	H	T	Q	A	T	N	D	P	P	E	V	T	V	F
*HLA-DPB1*04:01*	T	E	L	G	R	P	A	A	E	Y	W	N	S	Q	K	D	I	L	E	E	K	R	A	V	P	D	R	M	C	R	H	N	Y	E	L	G	G	P	M	T	L	Q	R	R
*MICA*008:01*	W	A	E	D	V	L	G	N	K	T	W	D	R	E	T	R	D	L	T	G	N	G	K	D	L	R	M	T	L	A	H	I	K	D	Q	K	E	G	L	H	S	L	Q	E

Figure S1. (Continued.)

HLA alleles*	HLA amino acid positions and residues																																											
	93	94	95	96	97	98	99	100	101	102	103	104	105	106	107	108	109	110	111	112	113	114	115	116	117	118	119	120	121	122	123	124	125	126	127	128	129	130	131	132	133	134	135	136
*HLA-A*02:01*	H	T	V	Q	R	M	Y	G	C	D	V	G	S	D	W	R	F	L	R	G	Y	H	Q	Y	A	Y	D	G	K	D	Y	I	A	L	K	E	D	L	R	S	W	T	A	A
*HLA-B*07:02*	H	T	L	Q	S	M	Y	G	C	D	V	G	P	D	G	R	L	L	R	G	H	D	Q	Y	A	Y	D	G	K	D	Y	I	A	L	N	E	D	L	R	S	W	T	A	A
*HLA-C*07:01*	H	T	L	Q	R	M	Y	G	C	D	L	G	P	D	G	R	L	L	R	G	Y	D	Q	S	A	Y	D	G	K	D	Y	I	A	L	N	E	D	L	R	S	W	T	A	A
*HLA-DRB1*07:01*	R	R	V	H	P	E	V	T	V	Y	P	A	K	T	Q	P	L	Q	H	H	N	L	L	V	C	S	V	S	G	F	Y	P	G	S	I	E	V	R	W	F	R	N	G	Q
*HLA-DQA1*05:01*	T	V	F	S	K	S	P	V	T	L	G	Q	P	N	I	L	I	C	L	V	D	N	I	F	P	P	V	V	N	I	T	W	L	S	N	G	H	S	V	T	E	G	V	S
*HLA-DQB1*03:01*	R	R	V	E	P	T	V	T	I	S	P	S	R	T	E	A	L	N	H	H	N	L	L	V	C	S	V	T	D	F	Y	P	A	Q	I	K	V	R	W	F	R	N	D	Q
*HLA-DPA1*01:03*	P	K	E	P	V	E	L	G	Q	P	N	T	L	I	C	H	I	D	K	F	F	P	P	V	L	N	V	T	W	L	C	N	G	E	L	V	T	E	G	V	A	E	S	L
*HLA-DPB1*04:01*	V	Q	P	R	V	N	V	S	P	S	K	K	G	P	L	Q	H	H	N	L	L	V	C	H	V	T	D	F	Y	P	G	S	I	Q	V	R	W	F	L	N	G	Q	E	E
*MICA*008:01*	I	R	V	C	E	I	H	E	D	N	S	T	R	S	S	Q	H	F	Y	Y	D	G	E	L	F	L	S	Q	N	L	E	T	E	E	W	T	V	P	Q	S	S	R	A	Q

HLA alleles*	HLA amino acid positions and residues																																											
	137	138	139	140	141	142	143	144	145	146	147	148	149	150	151	152	153	154	155	156	157	158	159	160	161	162	163	164	165	166	167	168	169	170	171	172	173	174	175	176	177	178	179	180
*HLA-A*02:01*	D	M	A	A	Q	T	T	K	H	K	W	E	A	A	H	V	A	E	Q	L	R	A	Y	L	E	G	T	C	V	E	W	L	R	R	Y	L	E	N	G	K	E	T	L	Q
*HLA-B*07:02*	D	T	A	A	Q	I	T	Q	R	K	W	E	A	A	R	E	A	E	Q	R	R	A	Y	L	E	G	E	C	V	E	W	L	R	R	Y	L	E	N	G	K	D	K	L	E
*HLA-C*07:01*	D	T	A	A	Q	I	T	Q	R	K	L	E	A	A	R	A	A	E	Q	L	R	A	Y	L	E	G	T	C	V	E	W	L	R	R	Y	L	E	N	G	K	E	T	L	Q
*HLA-DRB1*07:01*	E	E	K	A	G	V	V	S	T	G	L	I	Q	N	G	D	W	T	F	Q	T	L	V	M	L	E	T	V	P	R	S	G	E	V	Y	T	C	Q	V	E	H	P	S	V
*HLA-DQA1*05:01*	E	T	S	F	L	S	K	S	D	H	S	F	F	K	I	S	Y	L	T	L	L	P	S	A	E	E	S	Y	D	C	K	V	E	H	W	G	L	D	K	P	L	L	K	H
*HLA-DQB1*03:01*	E	E	T	T	G	V	V	S	T	P	L	I	R	N	G	D	W	T	F	Q	I	L	V	M	L	E	M	T	P	Q	H	G	D	V	Y	T	C	H	V	E	H	P	S	L
*HLA-DPA1*01:03*	F	L	P	R	T	D	Y	S	F	H	K	F	H	Y	L	T	F	V	P	S	A	E	D	F	Y	D	C	R	V	E	H	W	G	L	D	Q	P	L	L	K	H	W	E	A
*HLA-DPB1*04:01*	T	A	G	V	V	S	T	N	L	I	R	N	G	D	W	T	F	Q	I	L	V	M	L	E	M	T	P	Q	Q	G	D	V	Y	T	C	Q	V	E	H	T	S	L	D	S
*MICA*008:01*	T	L	A	M	N	V	R	N	F	L	K	E	D	A	M	K	T	K	T	H	Y	H	A	M	H	A	D	C	L	Q	E	L	R	R	Y	L	E	S	G	V	V	L	R	R

HLA alleles*	HLA amino acid positions and residues																																											
	181	182	183	184	185	186	187	188	189	190	191	192	193	194	195	196	197	198	199	200	201	202	203	204	205	206	207	208	209	210	211	212	213	214	215	216	217	218	219	220	221	222	223	224
*HLA-A*02:01*	R	T	D	A	P	K	T	H	M	T	H	H	A	V	S	D	H	E	A	T	L	R	C	W	A	L	S	F	Y	P	A	E	I	T	L	T	W	Q	R	D	G	E	D	Q
*HLA-B*07:02*	R	A	D	P	P	K	T	H	V	T	H	H	P	I	S	D	H	E	A	T	L	R	C	W	A	L	G	F	Y	P	A	E	I	T	L	T	W	Q	R	D	G	E	D	Q
*HLA-C*07:01*	R	A	E	P	P	K	T	H	V	T	H	H	P	L	S	D	H	E	A	T	L	R	C	W	A	L	G	F	Y	P	A	E	I	T	L	T	W	Q	R	D	G	E	D	Q
*HLA-DRB1*07:01*	M	S	P	L	T	V	E	W	R	A	R	S	E	S	A	Q	S	K	M	L	S	G	V	G	G	F	V	L	G	L	L	F	L	G	A	G	L	F	I	Y	F	R	N	Q
*HLA-DQA1*05:01*	W	E	P	E	I	P	A	P	M	S	E	L	T	E	T	V	V	C	A	L	G	L	S	V	G	L	V	G	I	V	V	G	T	V	F	I	I	R	G	L	R	S	V	G
*HLA-DQB1*03:01*	Q	N	P	I	T	V	E	W	R	A	Q	S	E	S	A	Q	S	K	M	L	S	G	I	G	G	F	V	L	G	L	I	F	L	G	L	G	L	I	I	H	H	R	S	Q
*HLA-DPA1*01:03*	Q	E	P	I	Q	M	P	E	T	T	E	T	V	L	C	A	L	G	L	V	L	G	L	V	G	I	I	V	G	T	V	L	I	I	K	S	L	R	S	G	H	D	P	R
*HLA-DPB1*04:01*	P	V	T	V	E	W	K	A	Q	S	D	S	A	R	S	K	T	L	T	G	A	G	G	F	V	L	G	L	I	I	C	G	V	G	I	F	M	H	R	R	S	K	K	V
*MICA*008:01*	T	V	P	P	M	V	N	V	T	R	S	E	A	S	E	G	N	I	T	V	T	C	R	A	S	S	F	Y	P	R	N	I	I	L	T	W	R	Q	D	G	V	S	L	S

Figure S1. (Continued.)

HLA alleles*	HLA amino acid positions and residues																																										
	225	226	227	228	229	230	231	232	233	234	235	236	237	238	239	240	241	242	243	244	245	246	247	248	249	250	251	252	253	254	255	256	257	258	259	260	261	262	263	264	265	266	267	268
*HLA-A*02:01*	T	Q	D	T	E	L	V	E	T	R	P	A	G	D	G	T	F	Q	K	W	A	A	V	V	V	P	S	G	Q	E	Q	R	Y	T	C	H	V	Q	H	E	G	L	P	K
*HLA-B*07:02*	T	Q	D	T	E	L	V	E	T	R	P	A	G	D	R	T	F	Q	K	W	A	A	V	V	V	P	S	G	E	E	Q	R	Y	T	C	H	V	Q	H	E	G	L	P	K
*HLA-C*07:01*	T	Q	D	T	E	L	V	E	T	R	P	A	G	D	G	T	F	Q	K	W	A	A	V	V	V	P	S	G	Q	E	Q	R	Y	T	C	H	M	Q	H	E	G	L	Q	E
*HLA-DRB1*07:01*	K	G	H	S	G	L	Q	P	T	G	F	L	S	x	x	x	x	x	x	x	x	x	x	x	x	x	x	x	x	x	x	x	x	x	x	x	x	x	x	x	x	x	x	x
*HLA-DQA1*05:01*	A	S	R	H	Q	G	P	L	x	x	x	x	x	x	x	x	x	x	x	x	x	x	x	x	x	x	x	x	x	x	x	x	x	x	x	x	x	x	x	x	x	x	x	x
*HLA-DQB1*03:01*	K	G	L	L	H	x	x	x	x	x	x	x	x	x	x	x	x	x	x	x	x	x	x	x	x	x	x	x	x	x	x	x	x	x	x	x	x	x	x	x	x	x	x	x
*HLA-DPA1*01:03*	A	Q	G	T	L	x	x	x	x	x	x	x	x	x	x	x	x	x	x	x	x	x	x	x	x	x	x	x	x	x	x	x	x	x	x	x	x	x	x	x	x	x	x	x
*HLA-DPB1*04:01*	Q	R	G	S	A	x	x	x	x	x	x	x	x	x	x	x	x	x	x	x	x	x	x	x	x	x	x	x	x	x	x	x	x	x	x	x	x	x	x	x	x	x	x	x
*MICA*008:01*	H	D	T	Q	Q	W	G	D	V	L	P	D	G	N	G	T	Y	Q	T	W	V	A	T	R	I	C	R	G	E	E	Q	R	F	T	C	Y	M	E	H	S	G	N	H	S

HLA alleles*	HLA amino acid positions and residues																																										
	269	270	271	272	273	274	275	276	277	278	279	280	281	282	283	284	285	286	287	288	289	290	291	292	293	294	295	296	297	298	299	300	301	302	303	304	305	306	307	308	309	310	311	312
*HLA-A*02:01*	P	L	T	L	R	W	E	P	S	S	Q	P	T	I	P	I	V	G	I	I	A	G	L	V	L	F	G	A	V	I	T	G	A	V	V	A	A	V	M	W	R	R	K	S
*HLA-B*07:02*	P	L	T	L	R	W	E	P	S	S	Q	S	T	V	P	I	V	G	I	V	A	G	L	A	V	L	A	V	V	V	I	G	A	V	V	A	A	V	M	C	R	R	K	S
*HLA-C*07:01*	P	L	T	L	S	W	E	P	S	S	Q	P	T	I	P	I	M	G	I	V	A	G	L	A	V	L	V	V	L	A	V	L	G	A	V	V	T	A	M	M	C	R	R	K
*HLA-DRB1*07:01*	x	x	x	x	x	x	x	x	x	x	x	x	x	x	x	x	x	x	x	x	x	x	x	x	x	x	x	x	x	x	x	x	x	x	x	x	x	x	x	x	x	x	x	x
*HLA-DQA1*05:01*	x	x	x	x	x	x	x	x	x	x	x	x	x	x	x	x	x	x	x	x	x	x	x	x	x	x	x	x	x	x	x	x	x	x	x	x	x	x	x	x	x	x	x	x
*HLA-DQB1*03:01*	x	x	x	x	x	x	x	x	x	x	x	x	x	x	x	x	x	x	x	x	x	x	x	x	x	x	x	x	x	x	x	x	x	x	x	x	x	x	x	x	x	x	x	x
*HLA-DPA1*01:03*	x	x	x	x	x	x	x	x	x	x	x	x	x	x	x	x	x	x	x	x	x	x	x	x	x	x	x	x	x	x	x	x	x	x	x	x	x	x	x	x	x	x	x	x
*HLA-DPB1*04:01*	x	x	x	x	x	x	x	x	x	x	x	x	x	x	x	x	x	x	x	x	x	x	x	x	x	x	x	x	x	x	x	x	x	x	x	x	x	x	x	x	x	x	x	x
*MICA*008:01*	T	H	P	V	P	S	G	K	V	L	V	L	Q	S	H	W	Q	T	F	H	V	S	A	V	A	A	G	C	Y	F	L	C	P	L	L	x	x	x	x	x	x	x	x	x

HLA alleles*	HLA amino acid positions and residues																																					
	313	314	315	316	317	318	319	320	321	322	323	324	325	326	327	328	329	330	331	332	333	334	335	336	337	338	339	340	341	342	343	344	345	346	347	348	349	350
*HLA-A*02:01*	S	D	R	K	G	G	S	Y	S	Q	A	A	S	S	D	S	A	Q	G	S	D	V	S	L	T	A	C	K	V	x	x	x	x	x	x	x	x	x
*HLA-B*07:02*	S	G	G	K	G	G	S	Y	S	Q	A	A	C	S	D	S	A	Q	G	S	D	V	S	L	T	A	x	x	x	x	x	x	x	x	x	x	x	x
*HLA-C*07:01*	S	S	G	G	K	G	G	S	C	S	Q	A	A	C	S	N	S	A	Q	G	S	D	E	S	L	I	T	C	K	A	x	x	x	x	x	x	x	x
*HLA-DRB1*07:01*	x	x	x	x	x	x	x	x	x	x	x	x	x	x	x	x	x	x	x	x	x	x	x	x	x	x	x	x	x	x	x	x	x	x	x	x	x	x
*HLA-DQA1*05:01*	x	x	x	x	x	x	x	x	x	x	x	x	x	x	x	x	x	x	x	x	x	x	x	x	x	x	x	x	x	x	x	x	x	x	x	x	x	x
*HLA-DQB1*03:01*	x	x	x	x	x	x	x	x	x	x	x	x	x	x	x	x	x	x	x	x	x	x	x	x	x	x	x	x	x	x	x	x	x	x	x	x	x	x
*HLA-DPA1*01:03*	x	x	x	x	x	x	x	x	x	x	x	x	x	x	x	x	x	x	x	x	x	x	x	x	x	x	x	x	x	x	x	x	x	x	x	x	x	x
*HLA-DPB1*04:01*	x	x	x	x	x	x	x	x	x	x	x	x	x	x	x	x	x	x	x	x	x	x	x	x	x	x	x	x	x	x	x	x	x	x	x	x	x	x
*MICA*008:01*	x	x	x	x	x	x	x	x	x	x	x	x	x	x	x	x	x	x	x	x	x	x	x	x	x	x	x	x	x	x	x	x	x	x	x	x	x	x

*: Amino acid sequences corresponding to the most common 4-digit alleles are indicated for the respective HLA genes and *MICA*.

For the definition of HLA gene amino acid positions, the start codon of the mature HLA protein was labeled as position 1, and the codon 5' to this was numbered position -1.

Figure S2. Stepwise regression analysis results of PsA-affected vs control individuals and PsC-affected vs control individuals.

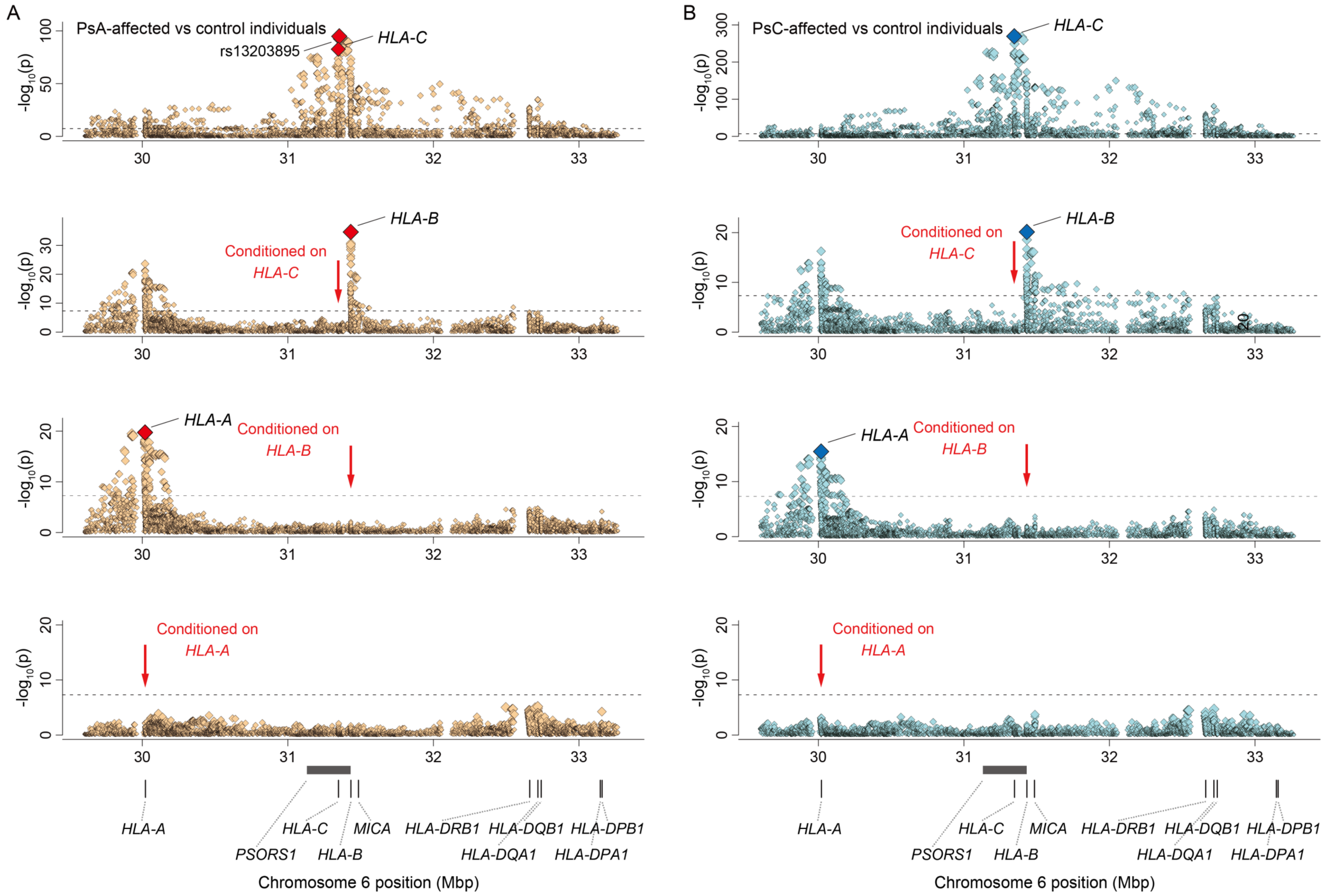

Stepwise conditional associations on (**A**) PsA-affected vs control individuals, (**B**) PsC-affected vs control individuals. Each diamond represents $-\log_{10}(p)$ of the variants, including SNPs, classical HLA or *MICA* alleles, and amino acid polymorphisms of the HLA genes or *MICA*. The dotted horizontal line represents the significance threshold of $P = 5.0\times10^{-8}$. The bottom panel shows the physical positions of the HLA genes, *MICA,* and *PSORS1* on chromosome 6 (UCSC Genome Browser hg18).

PsV; psoriasis vulgaris, PsA; psoriatic arthritis, PsC; cutaneous psoriasis.

Figure S3. Three-dimensional ribbon models for the HLA-A and HLA-DQ protein.

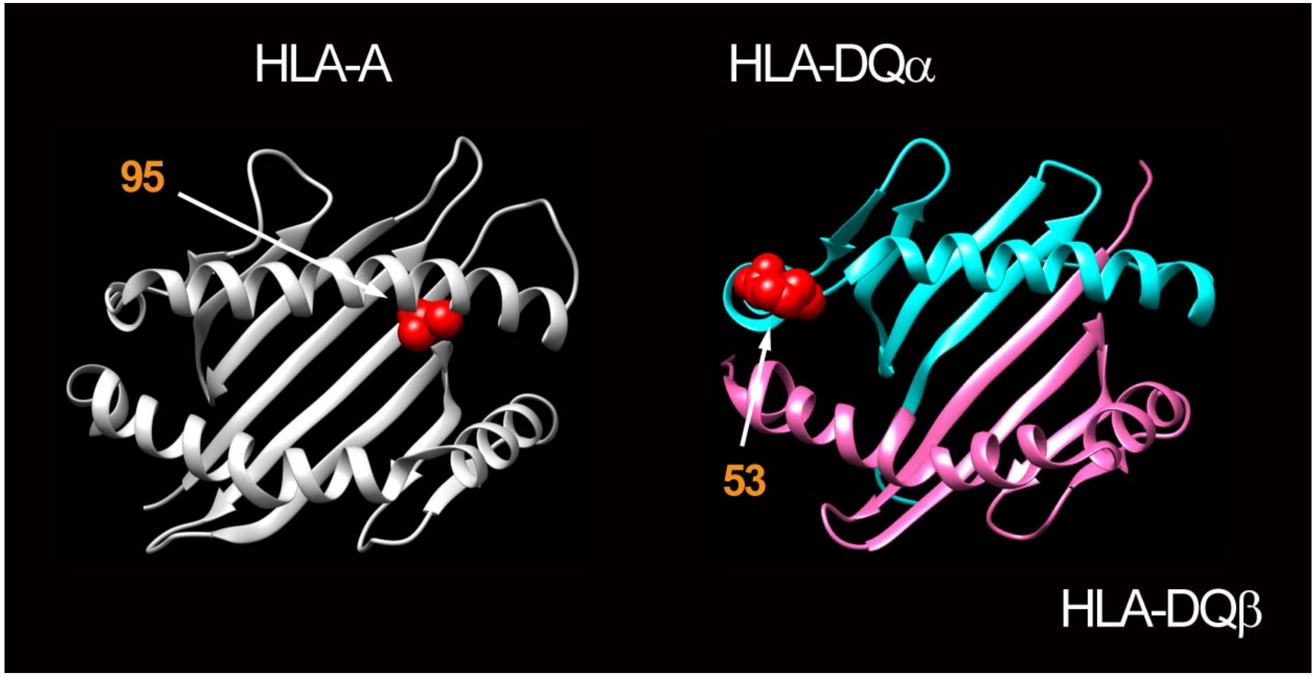

HLA-A and HLA-DQ protein structures are based on Protein Data Bank entries 1x7q and 1jk8, respectively, and prepared using UCSF Chimera version 1.7. Residues at amino acid positions overall PsV risk are highlighted as red spheres.

Figure S4. Linkage disequilibrium among risk HLA variants of class I HLA genes.

r^2 (obtained from European T1DGC reference panel)	HLA-A 95Ile	HLA-A 95Val	HLA-A 95Leu	*HLA-C*06:02*	*HLA-C*12:03*	HLA-B 67Tyr	HLA-B 67Phe	HLA-B 67Ser	HLA-B 67Cys	HLA-B 67Met	HLA-B 45Glu	HLA-B 45Thr	HLA-B 45Lys	HLA-B 45Met	HLA-B 45Gly	HLA-B 9Tyr	HLA-B 9Asp	HLA-B 9His
HLA-A 95Ile	-	-	-	0.00	0.00	0.00	0.02	0.03	0.00	0.00	0.02	0.00	0.02	0.02	0.00	0.01	0.03	0.00
HLA-A 95Val	-	-	-	0.00	0.00	0.00	0.02	0.02	0.00	0.00	0.01	0.01	0.01	0.02	0.00	0.01	0.02	0.00
HLA-A 95Leu	-	-	-	0.00	0.00	0.00	0.00	0.00	0.00	0.00	0.00	0.00	0.00	0.00	0.00	0.00	0.01	0.00
*HLA-C*06:02*	0.00	0.00	0.00	-	-	0.01	0.03	0.03	0.01	0.06	0.05	0.01	0.01	0.08	0.00	0.00	0.01	0.02
*HLA-C*12:03*	0.00	0.00	0.00	-	-	0.00	0.01	0.00	0.07	0.00	0.00	0.01	0.01	0.01	0.00	0.00	0.01	0.00
HLA-B 67Tyr	0.00	0.00	0.00	0.01	0.00	-	-	-	-	-	0.19	0.04	0.04	0.01	0.01	0.08	0.02	0.04
HLA-B 67Phe	0.02	0.02	0.00	0.03	0.01	-	-	-	-	-	0.03	0.11	0.11	0.06	0.00	0.03	0.43	0.12
HLA-B 67Ser	0.03	0.02	0.00	0.03	0.00	-	-	-	-	-	0.43	0.01	0.38	0.08	0.00	0.03	0.13	0.22
HLA-B 67Cys	0.00	0.00	0.00	0.01	0.07	-	-	-	-	-	0.21	0.04	0.04	0.02	0.00	0.00	0.02	0.00
HLA-B 67Met	0.00	0.00	0.00	0.06	0.00	-	-	-	-	-	0.03	0.02	0.01	0.06	0.00	0.03	0.01	0.02
HLA-B 45Glu	0.02	0.01	0.00	0.05	0.00	0.19	0.03	0.43	0.21	0.03	-	-	-	-	-	0.02	0.27	0.07
HLA-B 45Thr	0.00	0.01	0.00	0.01	0.01	0.04	0.11	0.01	0.04	0.02	-	-	-	-	-	0.01	0.06	0.01
HLA-B 45Lys	0.02	0.01	0.00	0.01	0.01	0.04	0.11	0.38	0.04	0.01	-	-	-	-	-	0.04	0.05	0.17
HLA-B 45Met	0.02	0.02	0.00	0.08	0.01	0.01	0.06	0.08	0.02	0.06	-	-	-	-	-	0.10	0.03	0.05
HLA-B 45Gly	0.00	0.00	0.00	0.00	0.00	0.01	0.00	0.00	0.00	0.00	-	-	-	-	-	0.00	0.00	0.00
HLA-B 9Tyr	0.01	0.01	0.00	0.00	0.00	0.08	0.03	0.03	0.00	0.03	0.02	0.01	0.04	0.10	0.00	-	-	-
HLA-B 9Asp	0.03	0.02	0.01	0.01	0.01	0.02	0.43	0.13	0.02	0.01	0.27	0.06	0.05	0.03	0.00	-	-	-
HLA-B 9His	0.00	0.00	0.00	0.02	0.00	0.04	0.12	0.22	0.00	0.02	0.07	0.01	0.17	0.05	0.00	-	-	-

Pairwise linkage disequilibrium matrix (r^2) values among psoriasis risk HLA variants of class I HLA genes, which were calculated based on the haplotype frequencies obtained the from T1DGC European reference panel.

Table S1. Characteristics of the data set collections.

Data set collections	Study design	No. psoriasis-affected individuals				No. control individuals	Total
		PsV	PsV clinical subtypes				
			PsA	PsC	PsA/PsC status unknown		
CASP	GWAS	1,339	340	693	306	1,374	2,713
Genizon		745	137	389	219	964	1,709
Kiel		463	32	269	162	1,130	1,593
PsA		1,414	1,414	0	0	1,385	2,847
CASP-DFU	Targeted follow-up study	1,563	133	575	855	1,141	2,704
PAGE	Immunochip	3,723	982	1,172	1,569	7,595	11,318
Total	-	9,247	3,038	3,098	3,111	13,589	22,884

PsV; psoriasis vulgaris, PsA; psoriatic arthritis, PsC; cutaneous psoriasis.

Table S2. Detailed stepwise association results of the HLA variants with psoriasis risk.

(A Microsoft Excel file for Table S2 is uploaded at the Journal web site.)

Table S3. Association results of the HLA variants on psoriasis risk.

HLA variant	Frequency				PsV-affected vs control individuals		PsA-affected vs control individuals		PsC-affected vs control individuals		PsA-affected vs PsC-affected individuals	
	PsV	PsA	PsC	Control	OR (95%CI)	*P*	OR (95%CI)	*P*	OR (95%CI)	*P*	OR (95%CI)	*P*
Multivaraite full regression model based on PsV-affected vs control individual analysis												
Classical *HLA-C* alleles												
*HLA-C*06:02*	0.23	0.19	0.28	0.093	3.26 (3.02-3.52)	2.1E-201	2.75 (2.43-3.12)	2.4E-56	2.75 (2.43-3.12)	5.9E-139	3.91 (3.52-4.35)	0.043
*HLA-C*12:03*	0.073	0.088	0.065	0.056	1.38 (1.26-1.52)	6.5E-12	1.50 (1.29-1.73)	9.7E-08	1.50 (1.29-1.73)	7.6E-07	1.42 (1.24-1.63)	0.33
Other *HLA-C* 4-digit alleles	0.70	0.72	0.66	0.85	(reference)		(reference)		(reference)		(reference)	
HLA-B amino acid position 67												
Cys	0.16	0.20	0.13	0.12	1.56 (1.45-1.67)	6.0E-35	2.01 (1.80-2.25)	2.8E-35	1.33 (1.19-1.49)	2.7E-07	1.53 (1.28-1.82)	2.0E-06
Met	0.12	0.10	0.14	0.046	1.44 (1.30-1.58)	2.6E-13	1.43 (1.22-1.67)	7.4E-06	1.53 (1.34-1.74)	2.1E-10	0.98 (0.81-1.20)	0.87
Tyr	0.12	0.13	0.11	0.16	1.00 (0.93-1.07)	0.93	1.13 (1.00-1.27)	0.048	0.93 (0.83-1.05)	0.24	1.26 (1.04-1.53)	0.016
Phe	0.21	0.21	0.21	0.26	1.00 (0.93-1.08)	0.99	0.98 (0.86-1.10)	0.70	1.03 (0.92-1.15)	0.60	0.95 (0.78-1.16)	0.63
Ser	0.39	0.36	0.41	0.42	(reference)		(reference)		(reference)		(reference)	
HLA-B amino acid position 9												
Asp	0.096	0.11	0.087	0.11	1.33 (1.21-1.45)	1.6E-09	1.64 (1.41-1.91)	1.0E-10	1.64 (1.41-1.91)	0.035	1.16 (1.01-1.34)	0.0010
Tyr	0.70	0.68	0.72	0.67	(reference)		(reference)		(reference)		(reference)	
His	0.20	0.21	0.19	0.22	0.87 (0.82-0.92)	1.6E-06	0.89 (0.81-0.98)	0.022	0.89 (0.81-0.98)	5.6E-06	0.81 (0.74-0.89)	0.48
HLA-A amino acid position 95												
Val	0.34	0.34	0.34	0.29	1.31 (1.25-1.38)	4.7E-28	1.46 (1.35-1.58)	2.9E-21	1.46 (1.35-1.58)	2.4E-10	1.26 (1.17-1.36)	0.13
Ile	0.56	0.56	0.56	0.59	(reference)		(reference)		(reference)		(reference)	
Leu	0.099	0.098	0.098	0.12	0.89 (0.83-0.95)	7.0E-04	0.94 (0.84-1.06)	0.32	0.94 (0.84-1.06)	0.0011	0.84 (0.75-0.93)	0.61
HLA-DQα1 amino acid position 53												
Arg	0.37	0.36	0.38	0.29	1.07 (1.01-1.13)	0.016	1.10 (1.01-1.20)	0.025	1.10 (1.01-1.20)	0.17	1.06 (0.98-1.14)	0.095
Lys	0.39	0.39	0.38	0.43	(reference)		(reference)		(reference)		(reference)	
Gln	0.25	0.25	0.24	0.29	0.91 (0.86-0.96)	9.3E-04	0.89 (0.81-0.98)	0.013	0.89 (0.81-0.98)	0.097	0.93 (0.85-1.01)	0.89
Association analysis based on PsA-affected vs PsC-affected individual analysis												
HLA-B amino acid position 45												
Glu	0.38	0.43	0.33	0.39	0.90 (0.86-0.94)	4.5E-07	1.14 (1.06-1.22)	1.8E-04	0.76 (0.71-0.81)	1.1E-18	1.46 (1.31-1.62)	2.9E-12
Thr, Lys, Met	0.62	0.57	0.67	0.61	(reference)		(reference)		(reference)		(reference)	

PsV; psoriasis vulgaris, PsA; psoriatic arthritis, PsC; cutaneous psoriasis, OR; odds ratio.

Table S4. Classical 4-digit *HLA-B* alleles corresponding to HLA-B amino acid residues at position 45.

HLA-B amino acid position 45	Classical 4-digit *HLA-B* alleles
Glu	**07:02, *07:05, *08:01, *14:01, *14:02, *15:03, *15:18, *27:02, *27:05, *38:01, *39:01, *39:06, *55:01, *56:01.*
Met	**13:02, *15:01, *15:17, *57:01.*
Lys	**40:01, *40:02, *41:01, *41:02, *44:02, *44:03, *44:05, *45:01, *47:01, *49:01, *50:01.*
Thr	**18:01, *35:01, *35:02, *35:03, *35:08, *37:01, *51:01, *52:01, *53:01, *58:01.*
