# Supplementary material for: Fine Mapping Major Histocompatibility Complex Associations in Psoriasis and Its Clinical Subtypes
Source: Am J Hum Genet. 2014 Aug 7;95(2):162–72. doi: 10.1016/j.ajhg.2014.07.002 (PMC4129407; doi:10.1016/j.ajhg.2014.07.002)
Supplement: Document S2. Article plus Supplemental Data [file mmc3.pdf]

ARTICLE

Fine Mapping Major Histocompatibility Complex Associations in Psoriasis and Its Clinical Subtypes

Yukinori Okada,[1,2,3,4,5,21] Buhm Han,[3,4,5,21] Lam C. Tsoi,[6] Philip E. Stuart,[7] Eva Ellinghaus,[8] Trilokraj Tejasvi,[7] Vinod Chandran,[9,10] Fawnda Pellett,[10] Remy Pollock,[10] Anne M. Bowcock,[11] Gerald G. Krueger,[12] Michael Weichenthal,[13] John J. Voorhees,[7] Proton Rahman,[14] Peter K. Gregersen,[15] Andre Franke,[8] Rajan P. Nair,[7] Gonçalo R. Abecasis,[6] Dafna D. Gladman,[9,10,16] James T. Elder,[7,17] Paul I.W. de Bakker,[18,19,*] and Soumya Raychaudhuri[3,4,5,20,*]

Psoriasis vulgaris (PsV) risk is strongly associated with variation within the major histocompatibility complex (MHC) region, but its genetic architecture has yet to be fully elucidated. Here, we conducted a large-scale fine-mapping study of PsV risk in the MHC region in 9,247 PsV-affected individuals and 13,589 controls of European descent by imputing class I and II human leukocyte antigen (HLA) genes from SNP genotype data. In addition, we imputed sequence variants for *MICA*, an MHC HLA-like gene that has been associated with PsV, to evaluate association at that locus as well. We observed that *HLA-C*06:02* demonstrated the lowest p value for overall PsV risk ($p = 1.7 \times 10^{-364}$). Stepwise analysis revealed multiple *HLA-C*06:02*-independent risk variants in both class I and class II HLA genes for PsV susceptibility (*HLA-C*12:03*, HLA-B amino acid positions 67 and 9, HLA-A amino acid position 95, and HLA-DQα1 amino acid position 53; $p < 5.0 \times 10^{-8}$), but no apparent risk conferred by *MICA*. We further evaluated risk of two major clinical subtypes of PsV, psoriatic arthritis (PsA; n = 3,038) and cutaneous psoriasis (PsC; n = 3,098). We found that risk heterogeneity between PsA and PsC might be driven by HLA-B amino acid position 45 ($p_{omnibus} = 2.2 \times 10^{-11}$), indicating that different genetic factors underlie the overall risk of PsV and the risk of specific PsV subphenotypes. Our study illustrates the value of high-resolution HLA and *MICA* imputation for fine mapping causal variants in the MHC.

Introduction

Psoriasis vulgaris (PsV [MIM 177900]) is a common immune-mediated skin disease characterized by epidermal hyperplasia, inflammatory cell infiltration, and vascular remodeling.[1,2] Approximately one-third of PsV-affected individuals develop a related inflammatory musculoskeletal condition called psoriatic arthritis (PsA), which is considered more severe than the other more common PsV subtype, cutaneous psoriasis (PsC).[3] Previous linkage and association studies have mapped PsV risk to a critical region spanning ~300 kb within the major histocompatibility complex (MHC) class I region on 6p21 (this region is termed *PSORS1*).[4] Subsequent analyses have identified *HLA-Cw6* (MIM 142840) as the risk allele of *PSORS1*.[5,6] Although recent large-scale genome-wide association studies (GWASs) have identified more than 35 genetic risk loci for PsV outside the MHC region, *HLA-C*06:02* (the most frequent four-digit allele equivalent to *HLA-Cw6*) has consistently demonstrated the strongest association with PsV risk.[7–12]

However, the effects of the genetic architecture of the MHC region on PsV risk have yet to be fully elucidated. Previous studies have suggested the existence of other risk variants in addition to *HLA-C*06:02* in class I human leukocyte antigen (HLA) genes (*HLA-A* [MIM 142800] or *HLA-B* [MIM 142830]) and class II HLA genes (*HLA-DRB1* [MIM 142857], *HLA-DQA1* [MIM 146880], *HLA-DQB1* [MIM 604305], *HLA-DPA1* [MIM 142880], or *HLA-DPB1* [MIM 142858]).[13–18] Investigators have also studied polymorphisms of MHC class I polypeptide-related sequence A (*MICA* [MIM 600169]), an HLA-like gene that does not present antigen.[17,19,20] However, strong and complex linkage-disequilibrium (LD) patterns in the MHC region[21,22] have thus far challenged the

[1]Department of Human Genetics and Disease Diversity, Graduate School of Medical and Dental Sciences, Tokyo Medical and Dental University, Tokyo 113-0085, Japan; [2]Laboratory for Statistical Analysis, RIKEN Center for Integrative Medical Sciences, Yokohama 230-0045, Japan; [3]Division of Rheumatology, Immunology, and Allergy, Brigham and Women's Hospital and Harvard Medical School, Boston, MA 02115, USA; [4]Division of Genetics, Brigham and Women's Hospital and Harvard Medical School, Boston, MA 02115, USA; [5]Program in Medical and Population Genetics, Broad Institute, Cambridge, MA 02142, USA; [6]Department of Biostatistics and Center for Statistical Genetics, University of Michigan, Ann Arbor, MI 48109, USA; [7]Department of Dermatology, University of Michigan Medical School, Ann Arbor, MI 48109, USA; [8]Institute of Clinical Molecular Biology, Kiel University, Kiel 24105, Germany; [9]Division of Rheumatology, Department of Medicine, University of Toronto, Toronto, ON M5T 2S8, Canada; [10]Centre for Prognosis Studies in the Rheumatic Diseases, Toronto Western Research Institute, University of Toronto, Toronto, ON M5T 2S8, Canada; [11]National Heart and Lung Institute, Imperial College, London SW7 2AZ, UK; [12]Department of Dermatology, University of Utah, Salt Lake City, UT 84112, USA; [13]Department of Dermatology, Christian-Albrechts-Universität zu Kiel, Kiel 24105, Germany; [14]Memorial University of Newfoundland, St. John's, NL A1C5S7, Canada; [15]The Feinstein Institute for Medical Research, North Shore – Long Island Jewish Health System, Manhasset, NY 11030, USA; [16]Toronto Western Research Institute, University of Toronto, Toronto, ON M5G 2M9, Canada; [17]Ann Arbor Veterans Affairs Hospital, Ann Arbor, MI 48105, USA; [18]Department of Medical Genetics, Center for Molecular Medicine, University Medical Center Utrecht, Utrecht 3584 CG, the Netherlands; [19]Department of Epidemiology, Julius Center for Health Sciences and Primary Care, University Medical Center Utrecht, Utrecht 3584 CG, the Netherlands; [20]Arthritis Research UK Epidemiology Unit, Centre for Musculoskeletal Research, Institute of Inflammation and Repair, University of Manchester, Manchester M13 9PT, UK
[21]These authors contributed equally to this work
*Correspondence: pdebakker@umcutrecht.nl (P.I.W.d.B.), soumya@broadinstitute.org (S.R.)
http://dx.doi.org/10.1016/j.ajhg.2014.07.002. ©2014 The Authors
This is an open access article under the CC BY license (http://creativecommons.org/licenses/by/3.0/).

identification of independent risk signals. Moreover, analyses focusing on the two major subsets of PsV have identified different effect sizes of associated risk alleles (including *HLA-C*06:02*) between PsA and PsC, suggesting heterogeneous effects of HLA alleles on the two major subphenotypes.[13–16,23]

Recently, we devised an analytical approach to fine map risk of the variants in the MHC region by imputing genotypes of amino acid polymorphisms in the classical HLA genes and classical two- and four-digit alleles.[24–26] Each classical four-digit HLA allele corresponds to a unique amino acid sequence encoded by the HLA gene, and thus polymorphic residues at each amino acid position could also be targets of disease-risk association studies. This approach has been used for successfully fine mapping HLA alleles of several autoimmune or immune-mediated diseases, including durable host control of HIV infection (MIM 609423), seropositive and seronegative rheumatoid arthritis (MIM 180300), myasthenia gravis (MIM 254200), and follicular lymphoma (MIM 613024).[25–29] In certain instances, our approach has been able to pinpoint individual amino acid sites that might account for disease risk within HLA molecules.[25–27]

In this study, we aimed to fully characterize the genetic architecture of the MHC region for PsV. Our goals were (1) to define the set of risk alleles for PsV at the four-digit HLA allele and amino acid resolutions, (2) to examine how the role of *MICA* in PsV compares to that of other HLA genes, and (3) to identify a genetic marker that distinguishes the risk of two subtypes, PsA and PsC. To this end, we applied our HLA-variant imputation approach to large-scale PsV GWASs and Immunochip studies comprising 9,247 affected individuals and 13,589 control individuals of European ancestry. We also expanded our approach to impute *MICA* alleles and MICA amino acid polymorphisms by constructing a *MICA* imputation reference panel. With the imputed MHC sequence variations, including classical HLA genes and *MICA*, we fine mapped the MHC associations with overall PsV risk and specifically focused on risk comparisons between the PsA and PsC subphenotypes.

Material and Methods

Samples

We used data from 9,247 PsV-affected individuals and 13,589 control individuals obtained from six case-control PsV data sets, including four GWASs (the Collaborative Association Study of Psoriasis [CASP] and the Genizon, Kiel, and PsA GWASs), a targeted deep follow-up study of CASP (the CASP-DFU), and one Immunochip-based data set of 3,723 affected and 7,595 control subjects (the Psoriasis Association Genetics Extension [PAGE] study), for a total of 9,247 affected and 13,589 control individuals (Table S1, available online).[7,9,12] Genotype data of the studies were generated and stringently quality-control (QC) filtered as described elsewhere,[7,9,12] and all samples were confirmed to be unrelated individuals of European ancestry according to self-reported ethnicity and results of principal-component (PC) analysis. All participating individuals provided written informed consent and were recruited according to the protocols approved by the institutional review board of each institution.

Phenotype Classification

All PsV-affected individuals were diagnosed by a dermatologist. Diagnosis of PsA was confirmed by a rheumatologist according to the Classification Criteria for Psoriatic Arthritis.[30] Individuals who had had PsV for 10 or more years but no signs of PsA were classified as having PsC. Our data set included 3,038 PsA subjects, 3,098 PsC subjects, and 3,111 subjects of unknown PsA or PsC status (Table S1).

Statistical Analysis

HLA Imputation

For each data set, we used SNP2HLA[24] to extract SNP genotypes located in the MHC region to impute classical two- and four-digit HLA alleles of and amino acid polymorphisms encoded by the eight class I and class II HLA genes (*HLA-A*, *HLA-B*, *HLA-C*, *HLA-DRB1*, *HLA-DQA1*, *HLA-DQB1*, *HLA-DPA1*, and *HLA-DPB1*). We conducted HLA imputation for each data set separately by using HLA and SNP genotypes from the Type 1 Diabetes Genetics Consortium (T1DGC; n = 5,225), which has demonstrated a high imputation accuracy for classical HLA alleles,[24,26,31] as a reference panel. We obtained information on HLA-gene polymorphisms from the IMGT/HLA Database.[32] Amino acid sequences encoded by the imputed HLA genes are indicated in Figure S1. For HLA amino acid positions, we indicate the start codon of the mature HLA protein as position 1, and we label the codon 5′ to this site as -1.[24] SNP2HLA checks concordance of allele strands of the A/T or G/C SNPs between the data set and the reference panel on the basis of allele-frequency comparison.[24] We applied postimputation QC criteria of MAF > 0.1% for the association analysis.

MICA Imputation

To expand our HLA imputation protocol into HLA-like genes, we constructed a reference panel for imputation of *MICA* variants. We obtained classical four-digit *MICA* alleles for the subjects from a subset of the PsA data set (n = 1,046). These samples were not selected in any particular way. We obtained MICA amino acid sequences from the IMGT/HLA Database[32] and the encoded MICA amino acid polymorphisms of the subjects, as well as the genotypes of *MICA* classical alleles and the genotyped SNPs in the MHC region. Using the constructed *MICA* reference panel and SNP2HLA,[24] we imputed *MICA* variants for the other data-set collections. Imputed genotypes of the *MICA* alleles and MICA amino acid polymorphisms were extracted and merged into those obtained from HLA imputation mentioned in the previous section. We empirically assessed the accuracy of imputing *MICA* variants by additionally genotyping *MICA* in a subset of the subjects from the PAGE Immunochip data set (n = 104) and comparing concordances of the imputed and genotyped classical *MICA* variants as described elsewhere.[24,26]

Statistical Framework for Association Analysis

We used the following analyses to test associations between HLA variants and risk of four binary phenotypes: (1) overall analysis of PsV susceptibility (PsV-affected versus control individuals), (2) stratified analysis of PsA susceptibility (PsA-affected versus control individuals), (3) stratified analysis of PsC susceptibility (PsC-affected versus control individuals), and (4) intra-PsV

analysis directly comparing PsA to PsC (PsA-affected versus PsC-affected individuals). For each phenotype, we assessed variant risk with a logistic-regression model assuming additive effects of the allele dosages in the log-odds scale and their fixed effects among the data-set collections. We defined HLA variants to include biallelic SNPs in the MHC region, two- and four-digit biallelic classical HLA or *MICA* alleles, biallelic HLA or MICA amino acid polymorphisms for respective residues, and multiallelic HLA or MICA amino acid polymorphisms for respective positions. To account for potential population-based and data-set-specific confounding factors, we included the top ten PCs and an indicator variable for each data set as covariates. For HLA variants with m alleles ($m = 2$ for biallelic variants and $m > 2$ for multiallelic variants), we included $m - 1$ alleles, excluding the most frequent allele as a reference, as independent variables in the regression model. This resulted in the following logistic-regression model:

$$\log(\text{odds}) = \beta_0 + \sum_{j=1}^{m-1} \beta_{1,j} x_j + \sum_{k=1}^{K} \left(\sum_{l=1}^{L} \beta_{2,k,l} y_{k,l} + \beta_{3,k} z_k \right) + \varepsilon,$$

where β_0 is the logistic-regression intercept and $\beta_{1,j}$ is the additive effect of the dosage of allele j for the variant x_j. K and L are numbers of the collections and PCs enrolled in the analysis. $y_{k,l}$ is the lth PC for the kth collection, and z_k is the indicator variable for the collection-specific intercept. $\beta_{2,k,l}$ and $\beta_{3,k}$ parameters are the effects of $y_{k,l}$ and z_k, respectively. An omnibus p value of the variant (p_{omnibus}) was obtained by a log-likelihood ratio test comparing the likelihood of the null model against the likelihood of the fitted model. We assessed the significance of the improvement in fit by calculating the deviance ($-2 \times$ the log likelihood ratio), which follows a χ^2 distribution with $m - 1$ degree(s) of freedom.

Conditional Association Analysis

For conditional association analysis, we considered the regression model including the additional HLA variants as covariates. When conditioning on specific HLA amino acid position(s), we included multiallelic variants of the amino acid residues as covariates. When conditioning on specific HLA gene(s), we included all two- and four-digit classical alleles of the HLA gene(s) (but not alleles with strong correlations [$R^2 > 0.97$]). We consecutively selected the HLA variants to be included as covariates for each HLA gene separately in a forward-type stepwise fashion until no variant satisfied the genome-wide significant threshold ($p < 5.0 \times 10^{-8}$). We tested a multivariate full regression model by including the *HLA-C*, *HLA-B*, *HLA-A*, and *HLA-DQA1* risk variants identified by the stepwise regression analysis as covariates and excluding the most frequent allele (or residue) from each locus (or amino acid position) as a reference allele (Table 1). Assuming a PsV prevalence of 2.0%, we estimated phenotypic variance explained by the risk HLA alleles and amino acid polymorphisms on the basis of the effect sizes obtained from the multivariate regression analysis and a liability threshold model.[2]

Testing for Discordant Effect Sizes on PsA and PsC

We tested whether the effect sizes of m classical four-digit alleles of the HLA gene had concordant risks between PsA and PsC, as described elsewhere.[26] For each of the two compared phenotypes (PsA-affected versus control individuals and PsC-affected versus control individuals), we calculated multivariate odds ratios (ORs) of $m - 1$ alleles by including them as binary independent variables in the regression model, where the most frequent allele was excluded as a reference. Let $\beta_{\text{PsA},1}, \ldots, \beta_{\text{PsA},m-1}$ and $v_{\text{PsA},1}, \ldots, v_{\text{PsA},m-1}$ be the multivariate log ORs and their variances, respectively, in PsA-affected versus control individuals, and let $\beta_{\text{PsC},1}, \ldots, \beta_{\text{PsC},m-1}$ and $v_{\text{PsC},1}, \ldots, v_{\text{PsC},m-1}$ be those in PsC-affected versus control individuals. We evaluated discordance of the effect sizes between the compared phenotypes ($p_{\text{heterogeneity}}$) by testing the statistic

$$\sum_{i=1}^{m-1} \frac{\left(\beta_{\text{PsA},i} - \beta_{\text{PsC},i}\right)^2}{v_{\text{PsA},i} + v_{\text{PsC},i}},$$

which follows a χ^2 distribution with $m - 1$ degrees of freedom under the null hypothesis of concordant effects.

Results

HLA and MICA Imputation

After imputation of HLA and *MICA*, we obtained genotypes for 7,078 SNPs in the MHC region (29.6–33.2 Mb at chromosome 6, UCSC Genome Browser hg18), 105 two-digit HLA or *MICA* alleles, 176 four-digit HLA or *MICA* alleles, and 438 amino acid polymorphisms encoded by HLA or *MICA* genes. Imputation of *MICA* demonstrated high concordance between genotyped and imputed genotypes (88.9% for both two- and four-digit alleles), which was comparable to that reported for imputation of other HLA genes.[24–26]

*HLA-C*06:02* Has the Strongest Association with PsV Risk

Unsurprisingly, when we tested the imputed variants in the MHC region for overall PsV risk (PsV-affected versus control individuals), the top association signal mapped to *HLA-C* (Figures 1A and 2A; Table S2). The most strongly associated variant was the classical *HLA-C*06:02* allele ($p = 1.7 \times 10^{-364}$), highly consistent with previous reports that *HLA-C*06:02* has the strongest association with PsV risk.[7–12,17,18] We observed that no HLA-C amino acid polymorphism was more strongly associated than *HLA-C*06:02* (the smallest p value, $p_{\text{omnibus}} = 4.5 \times 10^{-250}$, was at HLA-C amino acid position 156; Figure 3A). When conditioning on *HLA-C*06:02*, we observed the top association signal at the four-digit classical allele of *HLA-C*12:03* ($p = 2.5 \times 10^{-25}$). After conditioning on *HLA-C*06:02* and *HLA-C*12:03*, we found that no association exceeded the genome-wide significance threshold for the *HLA-C* variants ($p > 5.0 \times 10^{-8}$). These results suggest that multiple classical *HLA-C* alleles, exemplified by *HLA-C*06:02*, explain the influence of *HLA-C* on PsV risk.

PsV Risk Is Associated with Multiple Class I and Class II HLA Genes

We then investigated additional HLA-variant PsV risk independent of *HLA-C*. When we conditioned on all classical *HLA-C* alleles, we observed a significant independent association at HLA-B amino acid position 67 ($p_{\text{omnibus}} = 1.8 \times 10^{-45}$; Figures 2B and 3B). Stepwise

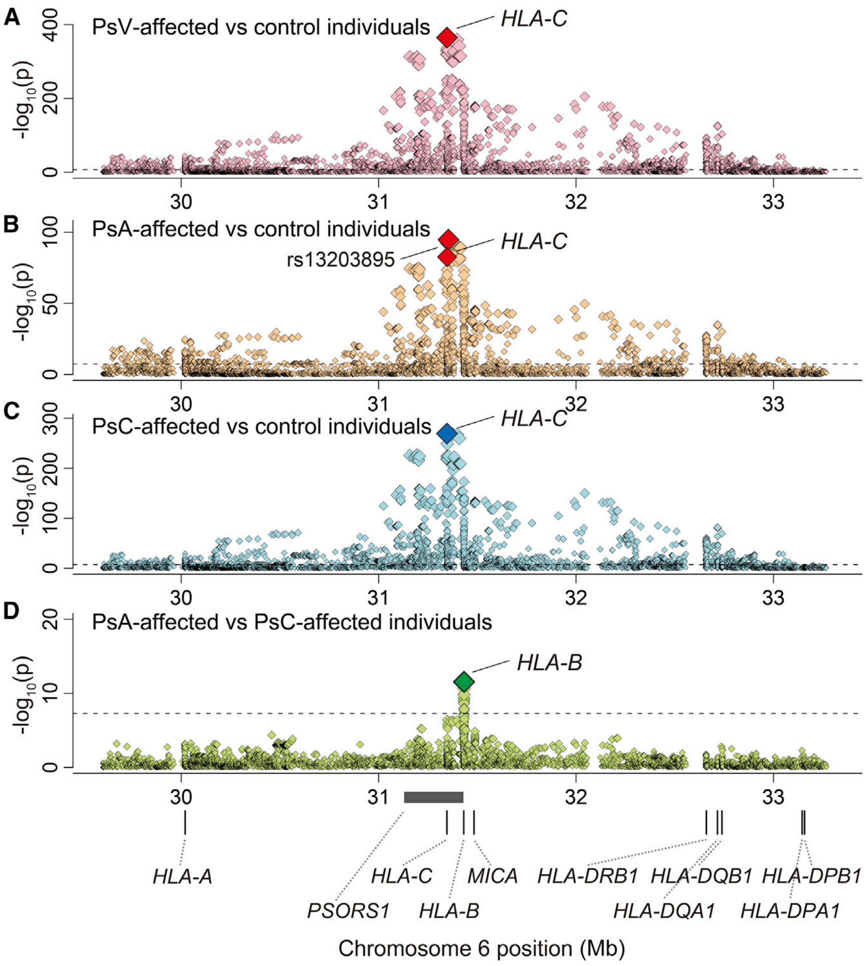

Figure 1. Plots of Nominal Association between the MHC Variants and PsV and Its Subphenotypes of PsA and PsC

Each diamond represents the $-\log_{10}(p)$ of the variants, including SNPs, classical HLA or *MICA* alleles, and amino acid polymorphisms encoded by the HLA genes or *MICA*. The dotted horizontal line represents the significance threshold of $p = 5.0 \times 10^{-8}$. The bottom panel shows the physical positions of the HLA genes, *MICA*, and *PSORS1* on chromosome 6 (UCSC Genome Browser hg18). We tested four binary phenotypes: (A) PsV-affected versus control individuals, (B) PsA-affected versus control individuals, (C) PsC-affected versus control individuals, and (D) PsA-affected versus PsC-affected individuals.

regression analysis of *HLA-B* variants further identified an independent association at position 9 ($p_{omnibus} = 1.5 \times 10^{-8}$). When we conditioned on *HLA-C* and *HLA-B*, we observed a significant independent association at HLA-A amino acid position 95 ($p_{omnibus} = 2.1 \times 10^{-36}$; Figures 2C and 3C). Stepwise analysis did not identify additional association within *HLA-A*. When we conditioned on the effects of *HLA-C*, *HLA-B*, and *HLA-A*, we observed a significant independent association at HLA-DQα1 amino acid position 53 ($p_{omnibus} = 4.2 \times 10^{-10}$; Figures 2D and 3D). Stepwise analysis did not identify an additional association within *HLA-DQA1*. When conditioning on *HLA-C*, *HLA-B*, *HLA-A*, and *HLA-DQA1*, we observed no other significant associations ($p > 5.0 \times 10^{-8}$; Figure 2E). These results demonstrate that PsV risk within the MHC region can be explained by combinations of multiple class I and class II HLA genes.

To highlight specific HLA alleles or amino acid residues that confer overall PsV risk, we defined a multivariate full regression model including all the identified risk variants of *HLA-C*, *HLA-B*, *HLA-A*, and *HLA-DQA1* (Table 1; Table S3). For *HLA-C*, we observed increased risk associated with *HLA-C*06:02* (OR = 3.26, 95% confidence interval [CI] = 3.02–3.52, $p = 2.1 \times 10^{-201}$) and *HLA-C*12:03* (OR = 1.38, 95% CI = 1.26–1.52, $p = 6.5 \times 10^{-12}$). For *HLA-B*, we observed increased risk associated with Cys67 (OR = 1.56, 95% CI = 1.45–1.67, $p = 6.0 \times 10^{-35}$), Met67 (OR = 1.44, 95% CI = 1.30–1.58, $p = 2.6 \times 10^{-13}$), and Asp9 (OR = 1.33, 95% CI = 1.21–1.45, $p = 1.6 \times 10^{-9}$). HLA-A Val95 (OR = 1.31, 95% CI = 1.25–1.38, $p = 4.7 \times 10^{-28}$) and HLA-DQα1 Arg53 (OR = 1.07, 95% CI = 1.01–1.13, p = 0.016) also demonstrated increased risk. In combination, these risk variants explained 6.7% of phenotype variance of PsV under the assumption of 2.0% of disease prevalence,[2] whereas *HLA-C*06:02* alone explained only 4.9% of the variance.

Risk Heterogeneity between PsA and PsC Is Explained by *HLA-B*

Next, we conducted an analysis focusing on subphenotypes of PsA and PsC. Overall, the association results of the PsA case-control analysis and of the PsC case-control analysis were similar to those of the PsV case-control analysis (Figures 1A–1C). The classical *HLA-C*06:02* allele demonstrated the lowest p value among the HLA variants in the MHC region. Stepwise association analyses, separately conducted for the PsA case-control analysis and the PsC case-control analysis, both revealed independent contributions of other class I HLA genes (*HLA-B* and *HLA-C*; Figure S2; Table S2). We did not observe independent signals for HLA class II genes in this stratified analysis, perhaps as a result of reduced statistical power.

Surprisingly, when we directly assessed comparative risk between PsA and PsC subjects, we found the lowest p value of the nominal association signal at HLA-B amino acid position 45 ($p_{omnibus} = 2.2 \times 10^{-11}$; Figures 1D and 2F) rather than at *HLA-C* alleles. After conditioning on HLA-B amino acid position 45, or all classical *HLA-B* alleles, we observed no significant association in the MHC region

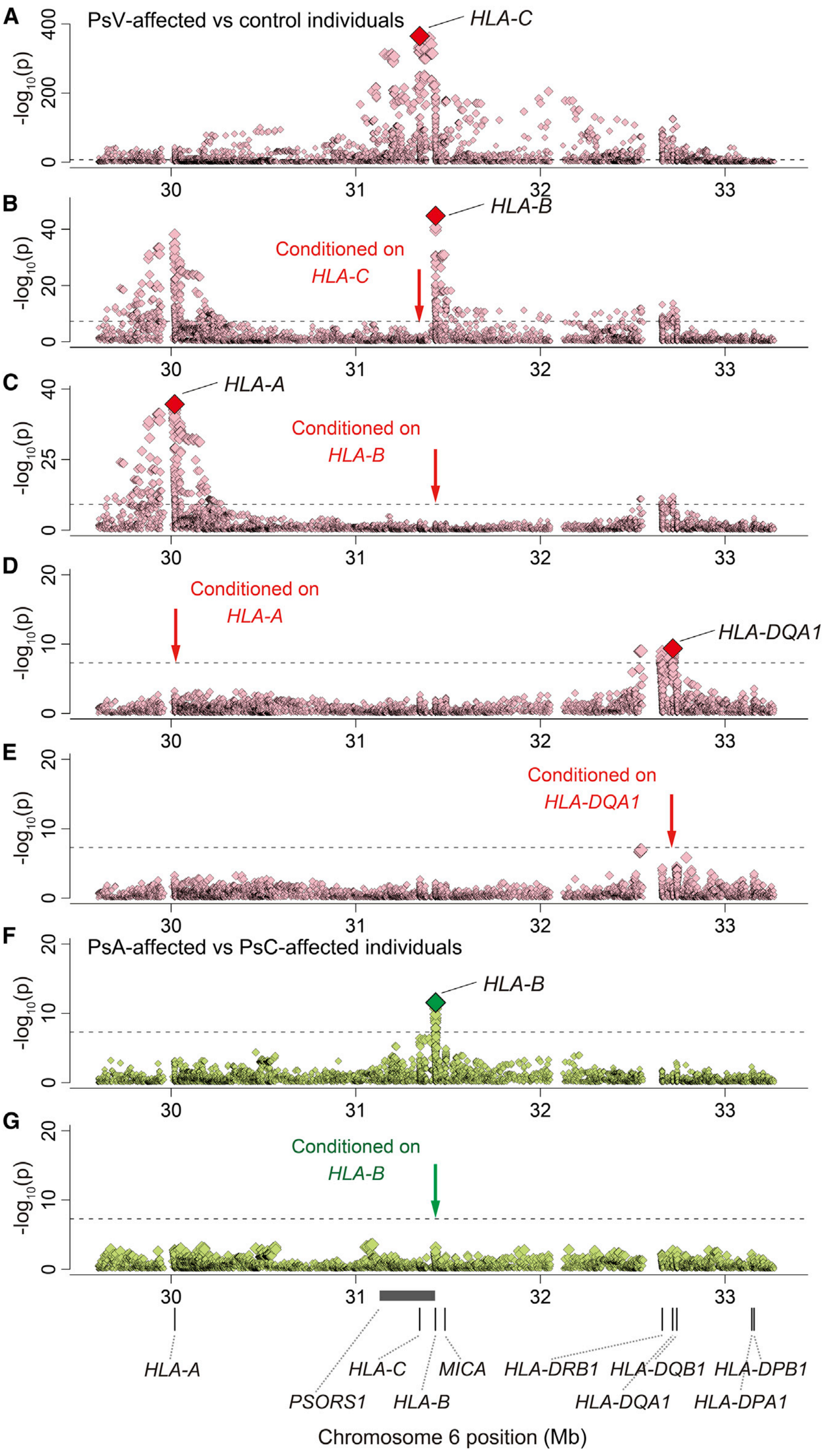

Figure 2. Plots of Stepwise Conditional Association of the Variants in the MHC Region

(A–E) Stepwise analysis of *HLA-C*, *HLA-B*, *HLA-A*, and *HLA-DQA1* in PsV-affected versus control individuals.

(F and G) Stepwise analysis of *HLA-B* in PsA versus PsC individuals. Each diamond represents the $-\log_{10}(p)$ of the variants, including SNPs, classical HLA or *MICA* alleles, and amino acid polymorphisms encoded by the HLA genes or *MICA*. The dotted horizontal line represents the significance threshold of $p = 5.0 \times 10^{-8}$. The most strongly associated amino acid polymorphisms and HLA classical alleles are labeled when their associations satisfied $p < 5.0 \times 10^{-8}$.

($p > 5.0 \times 10^{-8}$; Figures 2G and 3E). Of the HLA-B amino acid residues at position 45, HLA-B Glu45 increased PsA susceptibility in comparison to PsC susceptibility (OR = 1.46, 95% CI = 1.31–1.62, $p = 2.9 \times 10^{-12}$; Table 2; Table S2). Examining the classical alleles, we noted that *HLA-B*27*, a risk allele for another arthritic disease, ankylosing spondylitis (MIM 106300),[33] demonstrated the lowest p value for PsA versus PsC association ($p = 1.2 \times 10^{-4}$; Figure 4; Table S4) but much less significantly than HLA-B Glu45. We note that *HLA-B*27*, along with *HLA-B*38*, *HLA-B**39, and a number of other alleles, carries Glu at position 45.

Previous studies comparing PsA and PsC have suggested that *HLA-C* 06:02* shows increased PsC risk but decreased PsA risk,[13,14] and our study replicated this finding in a concordant directional effect ($p = 9.4 \times 10^{-6}$ for PsA versus PsC individuals). However, the differential impact of *HLA-C*06:02* on PsA and PsC risk disappeared after we conditioned on HLA-B amino acid position 45 (p = 0.12), suggesting that this reduced effect was the result of linkage to *HLA-B*. In contrast, the effect of HLA-B amino acid position 45 with respect to PsA versus PsC risk retained significance even after we conditioned on *HLA-C*06:02* ($p_{omnibus} = 2.2 \times 10^{-7}$).

We evaluated effect-size (OR) heterogeneity in classical four-digit alleles of the HLA genes between the two association analyses of PsA-affected versus control individuals and PsC-affected versus control individuals. Among the eight class I and class II HLA genes that we evaluated in the overall PsV case-control analysis, the *HLA-B* and *HLA-C* alleles showed significant risk heterogeneity ($p_{heterogeneity} = 5.8 \times 10^{-14}$ and $p_{heterogeneity} = 2.9 \times 10^{-6}$, respectively, with a significance threshold of $p < 0.05/8 = 0.0073$). When we conditioned on HLA-B amino acid position 45, risk heterogeneity diminished in both *HLA-B* and *HLA-C* classical alleles ($p_{heterogeneity} > 0.01$).

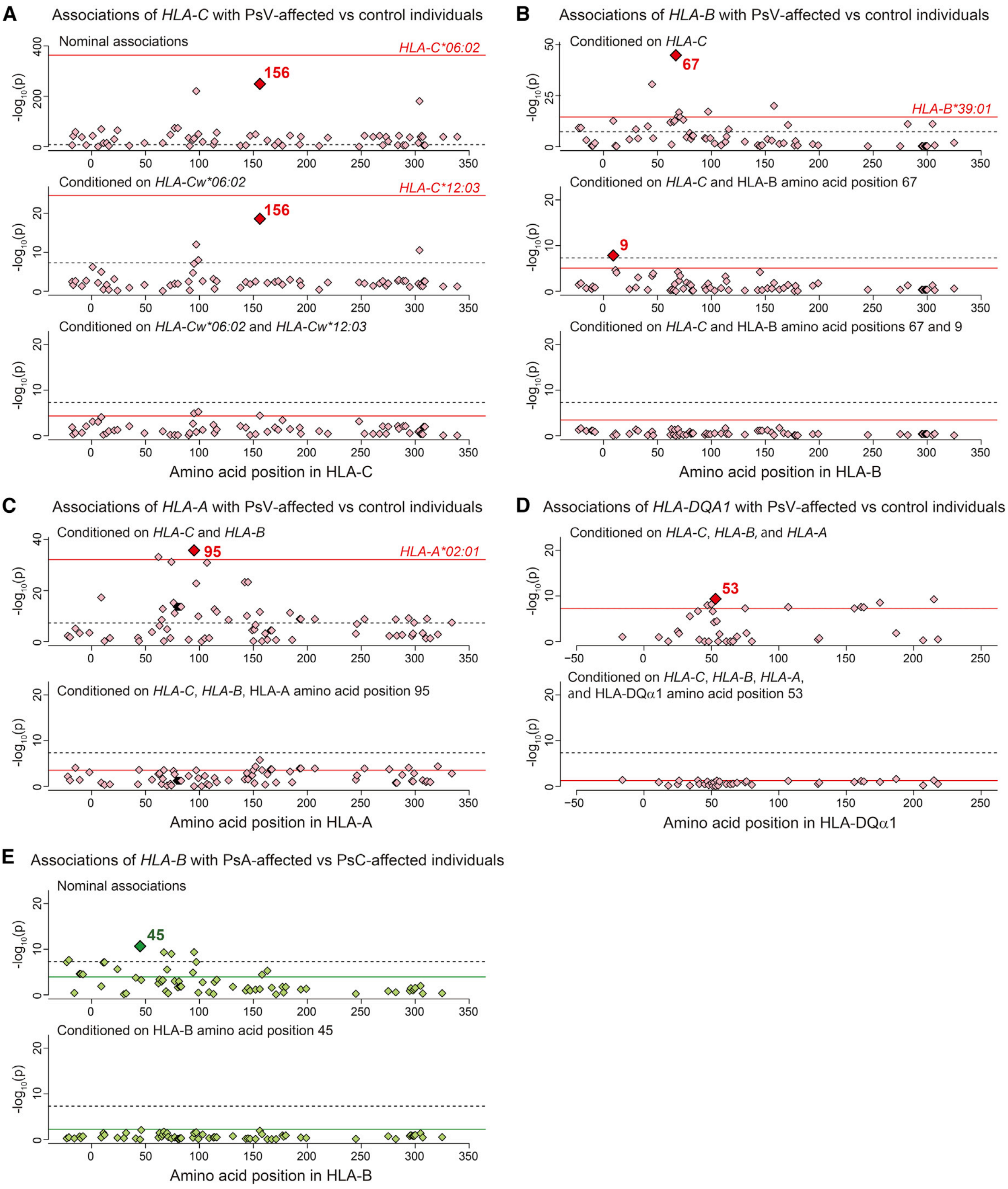

Figure 3. Plots of Stepwise Conditional Association of the HLA Amino Acid Polymorphisms and Classical HLA Alleles
(A–D) Stepwise analysis of *HLA-C*, *HLA-B*, *HLA-A*, and *HLA-DQA1* in PsV-affected versus control individuals.
(E) Stepwise analysis of *HLA-B* in PsA-affected versus PsC-affected individuals. Each diamond represents the $-\log_{10}(p_{omnibus})$ of the amino acid polymorphism encoded by the HLA gene. The colored horizontal line represents the $-\log_{10}(p_{omnibus})$ of the most strongly associated classical allele of the HLA gene. The dotted horizontal line represents the significance threshold of $p = 5.0 \times 10^{-8}$. The most strongly associated amino acid polymorphisms and HLA classical alleles are labeled when their associations satisfied $p < 5.0 \times 10^{-8}$.

In contrast, when we conditioned on *HLA-C*06:02*, risk heterogeneity diminished for the classical *HLA-C* alleles ($p_{heterogeneity} = 0.018$), but not in the classical *HLA-B* alleles ($p_{heterogeneity} = 6.5 \times 10^{-5}$). These results demonstrate that the risk heterogeneity between PsA and PsC primarily derives from *HLA-B*, but not *HLA-C* (or other), genes.

No Apparent Contribution of MICA to Psoriasis Risk

MICA variants demonstrated nominally significant associations with PsV, PsA, and PsC risk (the lowest p value, $p_{omnibus} = 2.2 \times 10^{-180}$, was at MICA amino acid position 298 for PsV-affected versus control individuals; Table S2). However, after we conditioned on *HLA-C* and *HLA-B*, no

Table 1. Association Results of the HLA Variants for Overall PsV Risk in Multivariate Regression Model

HLA Variant	Frequency		PsV-Affected versus Control Individuals	
	PsV	Control	OR (95% CI)[a]	p[a]
**Classical *HLA-C* Alleles**				
*HLA-C*06:02*	0.23	0.093	3.26 (3.02–3.52)	2.1×10^{-201}
*HLA-C*12:03*	0.073	0.056	1.38 (1.26–1.52)	6.5×10^{-12}
Other four-digit *HLA-C* alleles	0.70	0.85	reference	reference
**HLA-B Amino Acid Position 67**				
Cys	0.16	0.12	1.56 (1.45–1.67)	6.0×10^{-35}
Met	0.12	0.046	1.44 (1.30–1.58)	2.6×10^{-13}
Tyr	0.12	0.16	1.00 (0.93–1.07)	0.93
Phe	0.21	0.26	1.00 (0.93–1.08)	0.99
Ser	0.39	0.42	reference	reference
**HLA-B Amino Acid Position 9**				
Asp	0.096	0.11	1.33 (1.21–1.45)	1.6×10^{-9}
Tyr	0.70	0.67	reference	reference
His	0.20	0.22	0.87 (0.82–0.92)	1.6×10^{-6}
**HLA-A Amino Acid Position 95**				
Val	0.34	0.29	1.31 (1.25–1.38)	4.7×10^{-28}
Ile	0.56	0.59	reference	reference
Leu	0.099	0.12	0.89 (0.83–0.95)	7.0×10^{-4}
**HLA-DQα1 Amino Acid Position 53**				
Arg	0.37	0.29	1.07 (1.01–1.13)	0.016
Lys	0.39	0.43	reference	reference
Gln	0.25	0.29	0.91 (0.86–0.96)	9.3×10^{-4}

Abbreviations are as follows: CI, confidence interval; OR, odds ratio; and PsV, psoriasis vulgaris.
[a]Obtained from the multivariate full regression model including the *HLA-C*, *HLA-B*, *HLA-A*, and *HLA-DQA1* risk variants identified by the stepwise regression analysis.

MICA variants showed independent association signals ($p > 5.0 \times 10^{-8}$ for any of the four tested phenotype comparisons). Previous studies have suggested independent effects of several *MICA* variants, such as *MICA*016*, *MICA*008:01*, and MICA amino acid position 129, on PsV or its subphenotypes,[19,20] but our study did not observe apparent associations of these variants when we conditioned on *HLA-C* 06:02* or HLA-B amino acid position 67 ($p > 0.017$). We note that no significant association was observed in the *MICA* variants in direct comparison between PsA subjects and PsC subjects ($p > 5.0 \times 10^{-8}$). Therefore, our study could not identify an independent contribution of *MICA* on risk of psoriasis and its clinical subtypes.

Discussion

In this study, we fine mapped PsV risk within the MHC region. In addition to imputing variants of HLA genes, we evaluated risk of the HLA-like gene *MICA* by creating a *MICA* reference panel that empirically demonstrated high imputation accuracy. Our study identified multiple *HLA-C*06:02*-independent risk variants of both class I and class II HLA genes for PsV susceptibility (*HLA-B*, *HLA-A*, and *HLA-DQA1*), but no apparent risk attributable to *MICA*. We also observed that risk heterogeneity between PsA and PsC could be explained by polymorphisms of a single amino acid site encoded by *HLA-B*, suggesting that different genetic architectures underlie the overall risk of PsV and that of its subphenotypes. To our knowledge, ours is the largest HLA fine-mapping study of PsV associations in the MHC to date and defines genetic heterogeneity between PsA and PsC subphenotypes.

*HLA-C*06:02* has the strongest association with PsV risk, as reported previously.[7–12,17,18] We demonstrated that no single HLA-C amino acid polymorphism was more strongly associated than the *HLA-C*06:02* classical allele, suggesting that the haplotype sequence including *HLA-C*06:02* itself should be the origin of PsV risk. Several hypotheses might explain this. Clop et al. reported noncoding regulatory variants that are located in enhancer motifs and that are unique to the *HLA-C*06:02* haplotype.[34] A combination

Table 2. Association Results of HLA Variants for Subphenotype Risk Comparisons of PsA and PsC

HLA Variant	Frequency		PsA-Affected versus PsC-Affected Individuals	
	PsA	PsC	OR (95% CI)	p
**HLA-B Amino Acid Position 45 (Nominal)**				
Glu	0.43	0.33	1.46 (1.31–1.62)	2.9×10^{-12}
Thr, Lys, Met	0.57	0.67	reference	reference

Abbreviations are as follows: CI, confidence interval; OR, odds ratio; PsA, psoriatic arthritis; and PsC, cutaneous psoriasis.

of the polymorphisms in multiple HLA-C amino acid sites could effectively tag *HLA-C*06:02*.[35] We did observe a more modest independent effect at *HLA-C*12:03* in addition to the large *HLA-C*06:02* effect; Helms et al. reported that *HLA-C*06:02* and *HLA-C*12:03* share several functional domains and peptide-binding pockets of HLA-C.[5] We note that *HLA-C*12:03* did not show an independent association signal after we conditioned on *HLA-C*06:02* and every classical *HLA-B* allele ($p = 0.12$), suggesting the possibility that the observed *HLA-C*12:03* association might reflect risk at other *HLA-B* alleles in LD. Further functional studies will be necessary for elucidating the role of *HLA-C* in PsV risk.

In contrast, outside of *HLA-C*, amino acid polymorphisms (HLA-B amino acid positions 67 and 9, HLA-A position 95, and HLA-DQα1 position 53) demonstrated stronger associations than did classical alleles *HLA-B*, *HLA-A*, and *HLA-DQA1*. All of these amino acid sites were located within the HLA antigen binding (Figure 5 and Figure S3). Positions 67 and 9 in HLA-B have been identified in HLA fine-mapping studies for other immune-related diseases.[25–27] We note that LD structures between the amino acid positions could yield potential ambiguity in fine mapping of the causal amino acid position, and this might be clarified with larger studies.

The contribution of HLA-like genes to immune-related disease risk has long been a topic of discussion.[38,39] Although our imputation of *MICA* alleles was highly accurate, our study did not observe independent *MICA* risk of PsV after we conditioned on the neighboring risk HLA genes *HLA-C* and *HLA-B*. Previous studies focusing on *MICA* risk did not apply robust conditioning on all classical *HLA-C* and *HLA-B* alleles and thus could have potentially reflected the associations of *HLA-C* and *HLA-B* via LD with them.[19,20]

Here, we were able to successfully decompose the genetic architecture of PsA and PsC to a shared component and a subphenotype-specific component. Our study demonstrates that the HLA gene associated with the risk heterogeneity between PsA and PsC (*HLA-B*) is distinct from the HLA gene most associated with overall PsV risk (*HLA-C*). Previous studies have reported that *HLA-C*06:02* has different effect sizes for PsA and PsC, naturally leading to a hypothesis that the heterogeneity is driven by the difference in *HLA-C*, the major risk factor.[13–16,23] However, our observation is

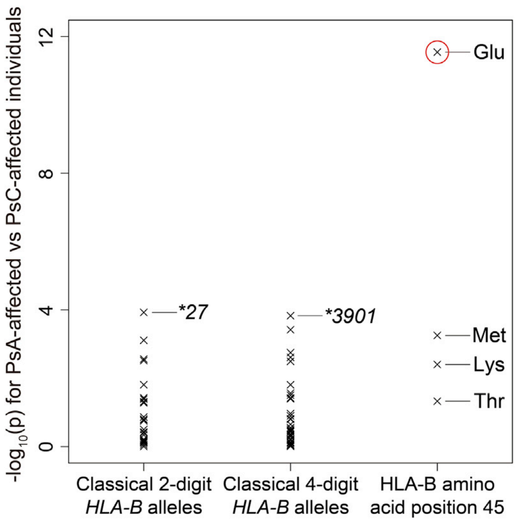

Figure 4. Associations of Classical *HLA-B* Alleles and HLA-B Amino Acid Position 45 for PsA versus PsC Individuals
The $-\log_{10}(p)$ of *HLA-B* classical two- and four-digit *HLA-B* alleles and HLA-B amino acid residues at position 45 in PsA versus PsC individuals. HLA-B Glu45 demonstrated the strongest association ($p = 2.9 \times 10^{-12}$; highlighted with a red circle), whereas the *HLA-B* classical alleles showed much smaller effects ($p > 1.0 \times 10^{-4}$).

more concordant with a model where the two PsV subtypes generally share the same risk alleles, including *HLA-C*06:02*, but differ at a specific locus that contributes to subtype differences.

HLA-B amino acid position 45 is the driving MHC position that modulates differential risk of PsA and PsV. The effect of Glu at HLA-B position 45 confers substantial risk of psoriatic arthritis and explains previously reported associations at *HLA-B*27*. This site is located within the binding groove of HLA-B and is classified as one of the functional pockets influencing receptor cell-surface expression or antigen peptide binding or presentation.[40] Being able to clinically distinguish those individuals who have isolated skin disease (PsC) from those who develop joint disease (PsA) has clinical importance. PsA often occurs in addition to psoriatic skin disease and almost always requires systemic therapy, for example, with anti-TNF or other biologic medications, for the prevention of destructive joint disease. In contrast, PsC can in many instances be managed with topical treatments alone.[3] Our findings might contribute to utilizing information on HLA variants to improve diagnostic approaches for clinical subphenotypes, as suggested for other complex diseases.[26] There is a possibility that the HLA-B45 amino acid residue tags other HLA-B driving risk variants at other amino acid sites, although we observed only limited LD between these sites (Figure S4). We also note that our method of evaluating OR heterogeneity (i.e., $p_{heterogeneity}$) might be conservative because of shared control subjects in the PsA and PsC case-control analyses. Further studies will be required for elucidating the functional mechanisms that result in differential PsA and PsC risk.

In summary, our study fine mapped risk of multiple class I and class II HLA genes in PsV and its subphenotypes

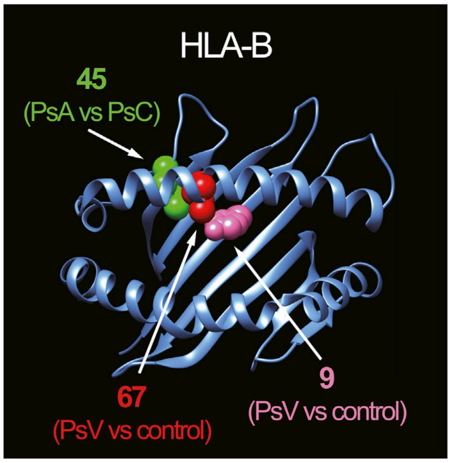

Figure 5. 3D Ribbon Models for HLA-B
HLA-B structures are based on Protein Data Bank entry 2bvp[36] and were prepared with UCSF Chimera version 1.7.[37] Amino acid position 67 and 9 residues associated with overall PsV risk are highlighted as red and pink spheres, respectively. An amino acid position 45 residue associated with subphenotype risk heterogeneity (PsA versus PsC) is highlighted as a green sphere.

through large-scale HLA and MICA imputation. Our study should contribute to our understanding of HLA variants in the etiology of PsV.

Supplemental Data

Supplemental Data include four figures and four tables and can be found with this article online at http://dx.doi.org/10.1016/j.ajhg.2014.07.002.

Acknowledgments

This work was supported by the NIH (1R01AR062886, R01AR042742, R01AR050511, R01AR062382, 5U01GM092691-04, 1R01AR063759-01A1, and 2R01AR050266), the Arthritis Foundation, a Clinical Scientist Development Award to S.R. from the Doris Duke Foundation, the Japan Society of the Promotion of Science, the Japan Science and Technology Agency, the Ann Arbor Veterans Affairs Hospital, the Canadian Institute of Health Research, the Krembil Foundation, the Arthritis Society, a Vernieuwingsimpuls VIDI Award (project 016.126.354) from the Netherlands Organization for Scientific Research, and the Babcock Memorial Trust.

Received: May 11, 2014
Accepted: July 7, 2014
Published: July 31, 2014

Web Resources

The URLs for data presented herein are as follows:

IMGT/HLA Database, http://www.ebi.ac.uk/ipd/imgt/hla/
Online Mendelian Inheritance in Man (OMIM), http://omim.org/
RefSeq, http://www.ncbi.nlm.nih.gov/refseq/
SNP2HLA, http://www.broadinstitute.org/mpg/snp2hla/
Type 1 Diabetes Genetics Consortium (T1DGC), https://www.t1dgc.org/home.cfm
UCSC Genome Browser, http://genome.ucsc.edu

References

1. Nestle, F.O., Kaplan, D.H., and Barker, J. (2009). Psoriasis. N. Engl. J. Med. *361*, 496–509.
2. Christophers, E. (2001). Psoriasis—epidemiology and clinical spectrum. Clin. Exp. Dermatol. *26*, 314–320.
3. Fitzgerald, O., and Winchester, R. (2009). Psoriatic arthritis: from pathogenesis to therapy. Arthritis Res. Ther. *11*, 214.
4. Veal, C.D., Capon, F., Allen, M.H., Heath, E.K., Evans, J.C., Jones, A., Patel, S., Burden, D., Tillman, D., Barker, J.N., and Trembath, R.C. (2002). Family-based analysis using a dense single-nucleotide polymorphism-based map defines genetic variation at PSORS1, the major psoriasis-susceptibility locus. Am. J. Hum. Genet. *71*, 554–564.
5. Helms, C., Saccone, N.L., Cao, L., Daw, J.A., Cao, K., Hsu, T.M., Taillon-Miller, P., Duan, S., Gordon, D., Pierce, B., et al. (2005). Localization of PSORS1 to a haplotype block harboring HLA-C and distinct from corneodesmosin and HCR. Hum. Genet. *118*, 466–476.
6. Nair, R.P., Stuart, P.E., Nistor, I., Hiremagalore, R., Chia, N.V., Jenisch, S., Weichenthal, M., Abecasis, G.R., Lim, H.W., Christophers, E., et al. (2006). Sequence and haplotype analysis supports HLA-C as the psoriasis susceptibility 1 gene. Am. J. Hum. Genet. *78*, 827–851.
7. Nair, R.P., Duffin, K.C., Helms, C., Ding, J., Stuart, P.E., Goldgar, D., Gudjonsson, J.E., Li, Y., Tejasvi, T., Feng, B.J., et al.; Collaborative Association Study of Psoriasis (2009). Genome-wide scan reveals association of psoriasis with IL-23 and NF-kappaB pathways. Nat. Genet. *41*, 199–204.
8. Strange, A., Capon, F., Spencer, C.C., Knight, J., Weale, M.E., Allen, M.H., Barton, A., Band, G., Bellenguez, C., Bergboer, J.G., et al.; Genetic Analysis of Psoriasis Consortium & the Wellcome Trust Case Control Consortium 2 (2010). A genome-wide association study identifies new psoriasis susceptibility loci and an interaction between HLA-C and ERAP1. Nat. Genet. *42*, 985–990.
9. Ellinghaus, E., Ellinghaus, D., Stuart, P.E., Nair, R.P., Debrus, S., Raelson, J.V., Belouchi, M., Fournier, H., Reinhard, C., Ding, J., et al. (2010). Genome-wide association study identifies a psoriasis susceptibility locus at TRAF3IP2. Nat. Genet. *42*, 991–995.
10. Hüffmeier, U., Uebe, S., Ekici, A.B., Bowes, J., Giardina, E., Korendowych, E., Juneblad, K., Apel, M., McManus, R., Ho, P., et al. (2010). Common variants at TRAF3IP2 are associated with susceptibility to psoriatic arthritis and psoriasis. Nat. Genet. *42*, 996–999.
11. Stuart, P.E., Nair, R.P., Ellinghaus, E., Ding, J., Tejasvi, T., Gudjonsson, J.E., Li, Y., Weidinger, S., Eberlein, B., Gieger, C., et al. (2010). Genome-wide association analysis identifies three psoriasis susceptibility loci. Nat. Genet. *42*, 1000–1004.
12. Tsoi, L.C., Spain, S.L., Knight, J., Ellinghaus, E., Stuart, P.E., Capon, F., Ding, J., Li, Y., Tejasvi, T., Gudjonsson, J.E., et al.; Collaborative Association Study of Psoriasis (CASP); Genetic Analysis of Psoriasis Consortium; Psoriasis Association Genetics Extension; Wellcome Trust Case Control Consortium 2 (2012). Identification of 15 new psoriasis susceptibility loci highlights the role of innate immunity. Nat. Genet. *44*, 1341–1348.
13. Eder, L., Chandran, V., Pellet, F., Shanmugarajah, S., Rosen, C.F., Bull, S.B., and Gladman, D.D. (2012). Human leucocyte antigen risk alleles for psoriatic arthritis among patients with psoriasis. Ann. Rheum. Dis. *71*, 50–55.

14. Eder, L., Chandran, V., Pellett, F., Shanmugarajah, S., Rosen, C.F., Bull, S.B., and Gladman, D.D. (2012). Differential human leucocyte allele association between psoriasis and psoriatic arthritis: a family-based association study. Ann. Rheum. Dis. *71*, 1361–1365.
15. Winchester, R., Minevich, G., Steshenko, V., Kirby, B., Kane, D., Greenberg, D.A., and FitzGerald, O. (2012). HLA associations reveal genetic heterogeneity in psoriatic arthritis and in the psoriasis phenotype. Arthritis Rheum. *64*, 1134–1144.
16. Chandran, V., Bull, S.B., Pellett, F.J., Ayearst, R., Rahman, P., and Gladman, D.D. (2013). Human leukocyte antigen alleles and susceptibility to psoriatic arthritis. Hum. Immunol. *74*, 1333–1338.
17. Feng, B.J., Sun, L.D., Soltani-Arabshahi, R., Bowcock, A.M., Nair, R.P., Stuart, P., Elder, J.T., Schrodi, S.J., Begovich, A.B., Abecasis, G.R., et al. (2009). Multiple Loci within the major histocompatibility complex confer risk of psoriasis. PLoS Genet. *5*, e1000606.
18. Knight, J., Spain, S.L., Capon, F., Hayday, A., Nestle, F.O., Clop, A., Barker, J.N., Weale, M.E., and Trembath, R.C.; Wellcome Trust Case Control Consortium; Genetic Analysis of Psoriasis Consortium; I-chip for Psoriasis Consortium (2012). Conditional analysis identifies three novel major histocompatibility complex loci associated with psoriasis. Hum. Mol. Genet. *21*, 5185–5192.
19. Pollock, R., Chandran, V., Barrett, J., Eder, L., Pellett, F., Yao, C., Lino, M., Shanmugarajah, S., Farewell, V.T., and Gladman, D.D. (2011). Differential major histocompatibility complex class I chain-related A allele associations with skin and joint manifestations of psoriatic disease. Tissue Antigens *77*, 554–561.
20. Pollock, R.A., Chandran, V., Pellett, F.J., Thavaneswaran, A., Eder, L., Barrett, J., Rahman, P., Farewell, V., and Gladman, D.D. (2013). The functional MICA-129 polymorphism is associated with skin but not joint manifestations of psoriatic disease independently of HLA-B and HLA-C. Tissue Antigens *82*, 43–47.
21. de Bakker, P.I., McVean, G., Sabeti, P.C., Miretti, M.M., Green, T., Marchini, J., Ke, X., Monsuur, A.J., Whittaker, P., Delgado, M., et al. (2006). A high-resolution HLA and SNP haplotype map for disease association studies in the extended human MHC. Nat. Genet. *38*, 1166–1172.
22. Okada, Y., Yamazaki, K., Umeno, J., Takahashi, A., Kumasaka, N., Ashikawa, K., Aoi, T., Takazoe, M., Matsui, T., Hirano, A., et al. (2011). HLA-Cw*1202-B*5201-DRB1*1502 haplotype increases risk for ulcerative colitis but reduces risk for Crohn's disease. Gastroenterology *141*, 864–871, e1–e5.
23. Ho, P.Y., Barton, A., Worthington, J., Plant, D., Griffiths, C.E., Young, H.S., Bradburn, P., Thomson, W., Silman, A.J., and Bruce, I.N. (2008). Investigating the role of the HLA-Cw*06 and HLA-DRB1 genes in susceptibility to psoriatic arthritis: comparison with psoriasis and undifferentiated inflammatory arthritis. Ann. Rheum. Dis. *67*, 677–682.
24. Jia, X., Han, B., Onengut-Gumuscu, S., Chen, W.M., Concannon, P.J., Rich, S.S., Raychaudhuri, S., and de Bakker, P.I. (2013). Imputing amino acid polymorphisms in human leukocyte antigens. PLoS ONE *8*, e64683.
25. Raychaudhuri, S., Sandor, C., Stahl, E.A., Freudenberg, J., Lee, H.S., Jia, X., Alfredsson, L., Padyukov, L., Klareskog, L., Worthington, J., et al. (2012). Five amino acids in three HLA proteins explain most of the association between MHC and seropositive rheumatoid arthritis. Nat. Genet. *44*, 291–296.
26. Han, B., Diogo, D., Eyre, S., Kallberg, H., Zhernakova, A., Bowes, J., Padyukov, L., Okada, Y., González-Gay, M.A., Rantapää-Dahlqvist, S., et al. (2014). Fine mapping seronegative and seropositive rheumatoid arthritis to shared and distinct HLA alleles by adjusting for the effects of heterogeneity. Am. J. Hum. Genet. *94*, 522–532.
27. Pereyra, F., Jia, X., McLaren, P.J., Telenti, A., de Bakker, P.I., Walker, B.D., Ripke, S., Brumme, C.J., Pulit, S.L., Carrington, M., et al.; International HIV Controllers Study (2010). The major genetic determinants of HIV-1 control affect HLA class I peptide presentation. Science *330*, 1551–1557.
28. Foo, J.N., Smedby, K.E., Akers, N.K., Berglund, M., Irwan, I.D., Jia, X., Li, Y., Conde, L., Darabi, H., Bracci, P.M., et al. (2013). Coding variants at hexa-allelic amino acid 13 of HLA-DRB1 explain independent SNP associations with follicular lymphoma risk. Am. J. Hum. Genet. *93*, 167–172.
29. Gregersen, P.K., Kosoy, R., Lee, A.T., Lamb, J., Sussman, J., McKee, D., Simpfendorfer, K.R., Pirskanen-Matell, R., Piehl, F., Pan-Hammarstrom, Q., et al. (2012). Risk for myasthenia gravis maps to a (151) Pro→Ala change in TNIP1 and to human leukocyte antigen-B*08. Ann. Neurol. *72*, 927–935.
30. Taylor, W., Gladman, D., Helliwell, P., Marchesoni, A., Mease, P., and Mielants, H.; CASPAR Study Group (2006). Classification criteria for psoriatic arthritis: development of new criteria from a large international study. Arthritis Rheum. *54*, 2665–2673.
31. Rich, S.S., Concannon, P., Erlich, H., Julier, C., Morahan, G., Nerup, J., Pociot, F., and Todd, J.A. (2006). The Type 1 Diabetes Genetics Consortium. Ann. N Y Acad. Sci. *1079*, 1–8.
32. Robinson, J., Halliwell, J.A., McWilliam, H., Lopez, R., Parham, P., and Marsh, S.G. (2013). The IMGT/HLA database. Nucleic Acids Res. *41* (Database issue), D1222–D1227.
33. Cortes, A., Hadler, J., Pointon, J.P., Robinson, P.C., Karaderi, T., Leo, P., Cremin, K., Pryce, K., Harris, J., Lee, S., et al.; International Genetics of Ankylosing Spondylitis Consortium (IGAS); Australo-Anglo-American Spondyloarthritis Consortium (TASC); Groupe Française d'Etude Génétique des Spondylarthrites (GFEGS); Nord-Trøndelag Health Study (HUNT); Spondyloarthritis Research Consortium of Canada (SPARCC); Wellcome Trust Case Control Consortium 2 (WTCCC2) (2013). Identification of multiple risk variants for ankylosing spondylitis through high-density genotyping of immune-related loci. Nat. Genet. *45*, 730–738.
34. Clop, A., Bertoni, A., Spain, S.L., Simpson, M.A., Pullabhatla, V., Tonda, R., Hundhausen, C., Di Meglio, P., De Jong, P., Hayday, A.C., et al. (2013). An in-depth characterization of the major psoriasis susceptibility locus identifies candidate susceptibility alleles within an HLA-C enhancer element. PLoS ONE *8*, e71690.
35. Asahina, A., Kuwata, S., Tokunaga, K., Juji, T., and Nakagawa, H. (1996). Study of aspartate at residue 9 of HLA-C molecules in Japanese patients with psoriasis vulgaris. J. Dermatol. Sci. *13*, 125–133.
36. Stewart-Jones, G.B., Gillespie, G., Overton, I.M., Kaul, R., Roche, P., McMichael, A.J., Rowland-Jones, S., and Jones, E.Y. (2005). Structures of three HIV-1 HLA-B*5703-peptide complexes and identification of related HLAs potentially associated with long-term nonprogression. J. Immunol. *175*, 2459–2468.
37. Pettersen, E.F., Goddard, T.D., Huang, C.C., Couch, G.S., Greenblatt, D.M., Meng, E.C., and Ferrin, T.E. (2004). UCSF

Chimera—a visualization system for exploratory research and analysis. J. Comput. Chem. *25*, 1605–1612.

38. Choy, M.K., and Phipps, M.E. (2010). MICA polymorphism: biology and importance in immunity and disease. Trends Mol. Med. *16*, 97–106.
39. Lo, P.H., Urabe, Y., Kumar, V., Tanikawa, C., Koike, K., Kato, N., Miki, D., Chayama, K., Kubo, M., Nakamura, Y., and Matsuda, K. (2013). Identification of a functional variant in the MICA promoter which regulates MICA expression and increases HCV-related hepatocellular carcinoma risk. PLoS ONE *8*, e61279.
40. Buxton, S.E., Benjamin, R.J., Clayberger, C., Parham, P., and Krensky, A.M. (1992). Anchoring pockets in human histocompatibility complex leukocyte antigen (HLA) class I molecules: analysis of the conserved B ("45") pocket of HLA-B27. J. Exp. Med. *175*, 809–820.

The American Journal of Human Genetics, Volume *95*

Supplemental Data

Fine Mapping Major Histocompatibility Complex Associations in Psoriasis and Its Clinical Subtypes

Yukinori Okada, Buhm Han, Lam C. Tsoi, Philip E. Stuart, Eva Ellinghaus, Trilokraj Tejasvi, Vinod Chandran, Fawnda Pellett, Remy Pollock, Anne M. Bowcock, Gerald G. Krueger, Michael Weichenthal, John J. Voorhees, Proton Rahman, Peter K. Gregersen, Andre Franke, Rajan P. Nair, Gonçalo R. Abecasis, Dafna D. Gladman, James T. Elder, Paul I.W. de Bakker, and Soumya Raychaudhuri

Figure S1. Amino acid sequences of HLA genes and *MICA*.

HLA alleles*	HLA amino acid positions and residues																																										
	-40	-39	-38	-37	-36	-35	-34	-33	-32	-31	-30	-29	-28	-27	-26	-25	-24	-23	-22	-21	-20	-19	-18	-17	-16	-15	-14	-13	-12	-11	-10	-9	-8	-7	-6	-5	-4	-3	-2	-1	1	2	3	4
*HLA-A*02:01*	x	x	x	x	x	x	x	x	x	x	x	x	x	x	x	x	M	A	V	M	A	P	R	T	L	V	L	L	L	S	G	A	L	A	L	T	Q	T	W	A	G	S	H	S
*HLA-B*07:02*	x	x	x	x	x	x	x	x	x	x	x	x	x	x	x	x	M	L	V	M	A	P	R	T	V	L	L	L	L	S	A	A	L	A	L	T	E	T	W	A	G	S	H	S
*HLA-C*07:01*	x	x	x	x	x	x	x	x	x	x	x	x	x	x	x	x	M	R	V	M	A	P	R	A	L	L	L	L	L	S	G	G	L	A	L	T	E	T	W	A	C	S	H	S
*HLA-DRB1*07:01*	x	x	x	x	x	x	x	x	x	x	x	M	V	C	L	K	L	P	G	G	S	C	M	A	A	L	T	V	T	L	M	V	L	S	S	P	L	A	L	A	G	D	T	Q
*HLA-DQA1*05:01*	x	x	x	x	x	x	x	x	x	x	x	x	x	x	x	x	x	M	I	L	N	K	A	L	M	L	G	A	L	A	L	T	T	V	M	S	P	C	G	G	E	D	I	V
*HLA-DQB1*03:01*	x	x	x	x	x	x	x	x	M	S	W	K	K	A	L	R	I	P	G	G	L	R	A	A	T	V	T	L	M	L	A	M	L	S	T	P	V	A	E	G	R	D	S	P
*HLA-DPA1*01:03*	x	x	x	x	x	x	x	x	x	M	R	P	E	D	R	M	F	H	I	R	A	V	I	L	R	A	L	S	L	A	F	L	L	S	L	R	G	A	G	A	I	K	A	D
*HLA-DPB1*04:01*	x	x	x	x	x	x	x	x	x	x	x	M	M	V	L	Q	V	S	A	A	P	R	T	V	A	L	T	A	L	L	M	V	L	L	T	S	V	V	Q	G	R	A	T	P
*MICA*008:01*	x	x	x	x	x	x	x	x	x	x	x	x	x	x	x	x	x	M	G	L	G	P	V	F	L	L	L	A	G	I	F	P	F	A	P	P	G	A	A	A	E	P	H	S

HLA alleles*	HLA amino acid positions and residues																																										
	5	6	7	8	9	10	11	12	13	14	15	16	17	18	19	20	21	22	23	24	25	26	27	28	29	30	31	32	33	34	35	36	37	38	39	40	41	42	43	44	45	46	47	48
*HLA-A*02:01*	M	R	Y	F	F	T	S	V	S	R	P	G	R	G	E	P	R	F	I	A	V	G	Y	V	D	D	T	Q	F	V	R	F	D	S	D	A	A	S	Q	R	M	E	P	R
*HLA-B*07:02*	M	R	Y	F	Y	T	S	V	S	R	P	G	R	G	E	P	R	F	I	S	V	G	Y	V	D	D	T	Q	F	V	R	F	D	S	D	A	A	S	P	R	E	E	P	R
*HLA-C*07:01*	M	R	Y	F	D	T	A	V	S	R	P	G	R	G	E	P	R	F	I	S	V	G	Y	V	D	D	T	Q	F	V	R	F	D	S	D	A	A	S	P	R	G	E	P	R
*HLA-DRB1*07:01*	P	R	F	L	W	Q	G	K	Y	K	C	H	F	F	N	G	T	E	R	V	Q	F	L	E	R	L	F	Y	N	Q	E	E	F	V	R	F	D	S	D	V	G	E	Y	R
*HLA-DQA1*05:01*	A	D	H	V	A	S	Y	G	V	N	L	Y	Q	S	Y	G	P	S	G	Q	Y	T	H	E	F	D	G	D	E	Q	F	Y	V	D	L	G	R	K	E	T	V	W	C	L
*HLA-DQB1*03:01*	E	D	F	V	Y	Q	F	K	A	M	C	Y	F	T	N	G	T	E	R	V	R	Y	V	T	R	Y	I	Y	N	R	E	E	Y	A	R	F	D	S	D	V	E	V	Y	R
*HLA-DPA1*01:03*	H	V	S	T	Y	A	A	F	V	Q	T	H	R	P	T	G	E	F	M	F	E	F	D	E	D	E	M	F	Y	V	D	L	D	K	K	E	T	V	W	H	L	E	E	F
*HLA-DPB1*04:01*	E	N	Y	L	F	Q	G	R	Q	E	C	Y	A	F	N	G	T	Q	R	F	L	E	R	Y	I	Y	N	R	E	E	F	A	R	F	D	S	D	V	G	E	F	R	A	V
*MICA*008:01*	L	R	Y	N	L	T	V	L	S	W	D	G	S	V	Q	S	G	F	L	A	E	V	H	L	D	G	Q	P	F	L	R	Y	D	R	Q	K	C	R	A	K	P	Q	G	Q

HLA alleles*	HLA amino acid positions and residues																																										
	49	50	51	52	53	54	55	56	57	58	59	60	61	62	63	64	65	66	67	68	69	70	71	72	73	74	75	76	77	78	79	80	81	82	83	84	85	86	87	88	89	90	91	92
*HLA-A*02:01*	A	P	W	I	E	Q	E	G	P	E	Y	W	D	G	E	T	R	K	V	K	A	H	S	Q	T	H	R	V	D	L	G	T	L	R	G	Y	Y	N	Q	S	E	A	G	S
*HLA-B*07:02*	A	P	W	I	E	Q	E	G	P	E	Y	W	D	R	N	T	Q	I	Y	K	A	Q	A	Q	T	D	R	E	S	L	R	N	L	R	G	Y	Y	N	Q	S	E	A	G	S
*HLA-C*07:01*	A	P	W	V	E	Q	E	G	P	E	Y	W	D	R	E	T	Q	N	Y	K	R	Q	A	Q	A	D	R	V	S	L	R	N	L	R	G	Y	Y	N	Q	S	E	D	G	S
*HLA-DRB1*07:01*	A	V	T	E	L	G	R	P	V	A	E	S	W	N	S	Q	K	D	I	L	E	D	R	R	G	Q	V	D	T	V	C	R	H	N	Y	G	V	G	E	S	F	T	V	Q
*HLA-DQA1*05:01*	P	V	L	R	Q	F	R	x	F	D	P	Q	F	A	L	T	N	I	A	V	L	K	H	N	L	N	S	L	I	K	R	S	N	S	T	A	A	T	N	E	V	P	E	V
*HLA-DQB1*03:01*	A	V	T	P	L	G	P	P	D	A	E	Y	W	N	S	Q	K	E	V	L	E	R	T	R	A	E	L	D	T	V	C	R	H	N	Y	Q	L	E	L	R	T	T	L	Q
*HLA-DPA1*01:03*	G	Q	A	F	S	F	E	A	Q	G	G	L	A	N	I	A	I	L	N	N	N	L	N	T	L	I	Q	R	S	N	H	T	Q	A	T	N	D	P	P	E	V	T	V	F
*HLA-DPB1*04:01*	T	E	L	G	R	P	A	A	E	Y	W	N	S	Q	K	D	I	L	E	E	K	R	A	V	P	D	R	M	C	R	H	N	Y	E	L	G	G	P	M	T	L	Q	R	R
*MICA*008:01*	W	A	E	D	V	L	G	N	K	T	W	D	R	E	T	R	D	L	T	G	N	G	K	D	L	R	M	T	L	A	H	I	K	D	Q	K	E	G	L	H	S	L	Q	E

Figure S1. (Continued.)

HLA alleles*	HLA amino acid positions and residues																																										
	93	94	95	96	97	98	99	100	101	102	103	104	105	106	107	108	109	110	111	112	113	114	115	116	117	118	119	120	121	122	123	124	125	126	127	128	129	130	131	132	133	134	135	136
*HLA-A*02:01*	H	T	V	Q	R	M	Y	G	C	D	V	G	S	D	W	R	F	L	R	G	Y	H	Q	Y	A	Y	D	G	K	D	Y	I	A	L	K	E	D	L	R	S	W	T	A	A
*HLA-B*07:02*	H	T	L	Q	S	M	Y	G	C	D	V	G	P	D	G	R	L	L	R	G	H	D	Q	Y	A	Y	D	G	K	D	Y	I	A	L	N	E	D	L	R	S	W	T	A	A
*HLA-C*07:01*	H	T	L	Q	R	M	Y	G	C	D	L	G	P	D	G	R	L	L	R	G	Y	D	Q	S	A	Y	D	G	K	D	Y	I	A	L	N	E	D	L	R	S	W	T	A	A
*HLA-DRB1*07:01*	R	R	V	H	P	E	V	T	V	Y	P	A	K	T	Q	P	L	Q	H	H	N	L	L	V	C	S	V	S	G	F	Y	P	G	S	I	E	V	R	W	F	R	N	G	Q
*HLA-DQA1*05:01*	T	V	F	S	K	S	P	V	T	L	G	Q	P	N	I	L	I	C	L	V	D	N	I	F	P	P	V	V	N	I	T	W	L	S	N	G	H	S	V	T	E	G	V	S
*HLA-DQB1*03:01*	R	R	V	E	P	T	V	T	I	S	P	S	R	T	E	A	L	N	H	H	N	L	L	V	C	S	V	T	D	F	Y	P	A	Q	I	K	V	R	W	F	R	N	D	Q
*HLA-DPA1*01:03*	P	K	E	P	V	E	L	G	Q	P	N	T	L	I	C	H	I	D	K	F	F	P	P	V	L	N	V	T	W	L	C	N	G	E	L	V	T	E	G	V	A	E	S	L
*HLA-DPB1*04:01*	V	Q	P	R	V	N	V	S	P	S	K	K	G	P	L	Q	H	H	N	L	L	V	C	H	V	T	D	F	Y	P	G	S	I	Q	V	R	W	F	L	N	G	Q	E	E
*MICA*008:01*	I	R	V	C	E	I	H	E	D	N	S	T	R	S	S	Q	H	F	Y	Y	D	G	E	L	F	L	S	Q	N	L	E	T	E	E	W	T	V	P	Q	S	S	R	A	Q

HLA alleles*	HLA amino acid positions and residues																																										
	137	138	139	140	141	142	143	144	145	146	147	148	149	150	151	152	153	154	155	156	157	158	159	160	161	162	163	164	165	166	167	168	169	170	171	172	173	174	175	176	177	178	179	180
*HLA-A*02:01*	D	M	A	A	Q	T	T	K	H	K	W	E	A	A	H	V	A	E	Q	L	R	A	Y	L	E	G	T	C	V	E	W	L	R	R	Y	L	E	N	G	K	E	T	L	Q
*HLA-B*07:02*	D	T	A	A	Q	I	T	Q	R	K	W	E	A	A	R	E	A	E	Q	R	R	A	Y	L	E	G	E	C	V	E	W	L	R	R	Y	L	E	N	G	K	D	K	L	E
*HLA-C*07:01*	D	T	A	A	Q	I	T	Q	R	K	L	E	A	A	R	A	A	E	Q	L	R	A	Y	L	E	G	T	C	V	E	W	L	R	R	Y	L	E	N	G	K	E	T	L	Q
*HLA-DRB1*07:01*	E	E	K	A	G	V	V	S	T	G	L	I	Q	N	G	D	W	T	F	Q	T	L	V	M	L	E	T	V	P	R	S	G	E	V	Y	T	C	Q	V	E	H	P	S	V
*HLA-DQA1*05:01*	E	T	S	F	L	S	K	S	D	H	S	F	F	K	I	S	Y	L	T	L	L	P	S	A	E	E	S	Y	D	C	K	V	E	H	W	G	L	D	K	P	L	L	K	H
*HLA-DQB1*03:01*	E	E	T	T	G	V	V	S	T	P	L	I	R	N	G	D	W	T	F	Q	I	L	V	M	L	E	M	T	P	Q	H	G	D	V	Y	T	C	H	V	E	H	P	S	L
*HLA-DPA1*01:03*	F	L	P	R	T	D	Y	S	F	H	K	F	H	Y	L	T	F	V	P	S	A	E	D	F	Y	D	C	R	V	E	H	W	G	L	D	Q	P	L	L	K	H	W	E	A
*HLA-DPB1*04:01*	T	A	G	V	V	S	T	N	L	I	R	N	G	D	W	T	F	Q	I	L	V	M	L	E	M	T	P	Q	Q	G	D	V	Y	T	C	Q	V	E	H	T	S	L	D	S
*MICA*008:01*	T	L	A	M	N	V	R	N	F	L	K	E	D	A	M	K	T	K	T	H	Y	H	A	M	H	A	D	C	L	Q	E	L	R	R	Y	L	E	S	G	V	V	L	R	R

HLA alleles*	HLA amino acid positions and residues																																										
	181	182	183	184	185	186	187	188	189	190	191	192	193	194	195	196	197	198	199	200	201	202	203	204	205	206	207	208	209	210	211	212	213	214	215	216	217	218	219	220	221	222	223	224
*HLA-A*02:01*	R	T	D	A	P	K	T	H	M	T	H	H	A	V	S	D	H	E	A	T	L	R	C	W	A	L	S	F	Y	P	A	E	I	T	L	T	W	Q	R	D	G	E	D	Q
*HLA-B*07:02*	R	A	D	P	P	K	T	H	V	T	H	H	P	I	S	D	H	E	A	T	L	R	C	W	A	L	G	F	Y	P	A	E	I	T	L	T	W	Q	R	D	G	E	D	Q
*HLA-C*07:01*	R	A	E	P	P	K	T	H	V	T	H	H	P	L	S	D	H	E	A	T	L	R	C	W	A	L	G	F	Y	P	A	E	I	T	L	T	W	Q	R	D	G	E	D	Q
*HLA-DRB1*07:01*	M	S	P	L	T	V	E	W	R	A	R	S	E	S	A	Q	S	K	M	L	S	G	V	G	G	F	V	L	G	L	L	F	L	G	A	G	L	F	I	Y	F	R	N	Q
*HLA-DQA1*05:01*	W	E	P	E	I	P	A	P	M	S	E	L	T	E	T	V	V	C	A	L	G	L	S	V	G	L	V	G	I	V	V	G	T	V	F	I	I	R	G	L	R	S	V	G
*HLA-DQB1*03:01*	Q	N	P	I	T	V	E	W	R	A	Q	S	E	S	A	Q	S	K	M	L	S	G	I	G	G	F	V	L	G	L	I	F	L	G	L	G	L	I	I	H	H	R	S	Q
*HLA-DPA1*01:03*	Q	E	P	I	Q	M	P	E	T	T	E	T	V	L	C	A	L	G	L	V	L	G	L	V	G	I	I	V	G	T	V	L	I	I	K	S	L	R	S	G	H	D	P	R
*HLA-DPB1*04:01*	P	V	T	V	E	W	K	A	Q	S	D	S	A	R	S	K	T	L	T	G	A	G	G	F	V	L	G	L	I	I	C	G	V	G	I	F	M	H	R	R	S	K	K	V
*MICA*008:01*	T	V	P	P	M	V	N	V	T	R	S	E	A	S	E	G	N	I	T	V	T	C	R	A	S	S	F	Y	P	R	N	I	I	L	T	W	R	Q	D	G	V	S	L	S

Figure S1. (Continued.)

HLA alleles*	HLA amino acid positions and residues																																										
	225	226	227	228	229	230	231	232	233	234	235	236	237	238	239	240	241	242	243	244	245	246	247	248	249	250	251	252	253	254	255	256	257	258	259	260	261	262	263	264	265	266	267	268
*HLA-A*02:01*	T	Q	D	T	E	L	V	E	T	R	P	A	G	D	G	T	F	Q	K	W	A	A	V	V	V	P	S	G	Q	E	Q	R	Y	T	C	H	V	Q	H	E	G	L	P	K
*HLA-B*07:02*	T	Q	D	T	E	L	V	E	T	R	P	A	G	D	R	T	F	Q	K	W	A	A	V	V	V	P	S	G	E	E	Q	R	Y	T	C	H	V	Q	H	E	G	L	P	K
*HLA-C*07:01*	T	Q	D	T	E	L	V	E	T	R	P	A	G	D	G	T	F	Q	K	W	A	A	V	V	V	P	S	G	Q	E	Q	R	Y	T	C	H	M	Q	H	E	G	L	Q	E
*HLA-DRB1*07:01*	K	G	H	S	G	L	Q	P	T	G	F	L	S	x	x	x	x	x	x	x	x	x	x	x	x	x	x	x	x	x	x	x	x	x	x	x	x	x	x	x	x	x	x	x
*HLA-DQA1*05:01*	A	S	R	H	Q	G	P	L	x	x	x	x	x	x	x	x	x	x	x	x	x	x	x	x	x	x	x	x	x	x	x	x	x	x	x	x	x	x	x	x	x	x	x	x
*HLA-DQB1*03:01*	K	G	L	L	H	x	x	x	x	x	x	x	x	x	x	x	x	x	x	x	x	x	x	x	x	x	x	x	x	x	x	x	x	x	x	x	x	x	x	x	x	x	x	x
*HLA-DPA1*01:03*	A	Q	G	T	L	x	x	x	x	x	x	x	x	x	x	x	x	x	x	x	x	x	x	x	x	x	x	x	x	x	x	x	x	x	x	x	x	x	x	x	x	x	x	x
*HLA-DPB1*04:01*	Q	R	G	S	A	x	x	x	x	x	x	x	x	x	x	x	x	x	x	x	x	x	x	x	x	x	x	x	x	x	x	x	x	x	x	x	x	x	x	x	x	x	x	x
*MICA*008:01*	H	D	T	Q	Q	W	G	D	V	L	P	D	G	N	G	T	Y	Q	T	W	V	A	T	R	I	C	R	G	E	E	Q	R	F	T	C	Y	M	E	H	S	G	N	H	S

HLA alleles*	HLA amino acid positions and residues																																										
	269	270	271	272	273	274	275	276	277	278	279	280	281	282	283	284	285	286	287	288	289	290	291	292	293	294	295	296	297	298	299	300	301	302	303	304	305	306	307	308	309	310	311	312
*HLA-A*02:01*	P	L	T	L	R	W	E	P	S	S	Q	P	T	I	P	I	V	G	I	I	A	G	L	V	L	F	G	A	V	I	T	G	A	V	V	A	A	V	M	W	R	R	K	S
*HLA-B*07:02*	P	L	T	L	R	W	E	P	S	S	Q	S	T	V	P	I	V	G	I	V	A	G	L	A	V	L	A	V	V	V	I	G	A	V	V	A	A	V	M	C	R	R	K	S
*HLA-C*07:01*	P	L	T	L	S	W	E	P	S	S	Q	P	T	I	P	I	M	G	I	V	A	G	L	A	V	L	V	V	L	A	V	L	G	A	V	V	T	A	M	M	C	R	R	K
*HLA-DRB1*07:01*	x	x	x	x	x	x	x	x	x	x	x	x	x	x	x	x	x	x	x	x	x	x	x	x	x	x	x	x	x	x	x	x	x	x	x	x	x	x	x	x	x	x	x	x
*HLA-DQA1*05:01*	x	x	x	x	x	x	x	x	x	x	x	x	x	x	x	x	x	x	x	x	x	x	x	x	x	x	x	x	x	x	x	x	x	x	x	x	x	x	x	x	x	x	x	x
*HLA-DQB1*03:01*	x	x	x	x	x	x	x	x	x	x	x	x	x	x	x	x	x	x	x	x	x	x	x	x	x	x	x	x	x	x	x	x	x	x	x	x	x	x	x	x	x	x	x	x
*HLA-DPA1*01:03*	x	x	x	x	x	x	x	x	x	x	x	x	x	x	x	x	x	x	x	x	x	x	x	x	x	x	x	x	x	x	x	x	x	x	x	x	x	x	x	x	x	x	x	x
*HLA-DPB1*04:01*	x	x	x	x	x	x	x	x	x	x	x	x	x	x	x	x	x	x	x	x	x	x	x	x	x	x	x	x	x	x	x	x	x	x	x	x	x	x	x	x	x	x	x	x
*MICA*008:01*	T	H	P	V	P	S	G	K	V	L	V	L	Q	S	H	W	Q	T	F	H	V	S	A	V	A	A	G	C	Y	F	L	C	P	L	L	x	x	x	x	x	x	x	x	x

HLA alleles*	HLA amino acid positions and residues																																					
	313	314	315	316	317	318	319	320	321	322	323	324	325	326	327	328	329	330	331	332	333	334	335	336	337	338	339	340	341	342	343	344	345	346	347	348	349	350
*HLA-A*02:01*	S	D	R	K	G	G	S	Y	S	Q	A	A	S	S	D	S	A	Q	G	S	D	V	S	L	T	A	C	K	V	x	x	x	x	x	x	x	x	x
*HLA-B*07:02*	S	G	G	K	G	G	S	Y	S	Q	A	A	C	S	D	S	A	Q	G	S	D	V	S	L	T	A	x	x	x	x	x	x	x	x	x	x	x	x
*HLA-C*07:01*	S	S	G	G	K	G	G	S	C	S	Q	A	A	C	S	N	S	A	Q	G	S	D	E	S	L	I	T	C	K	A	x	x	x	x	x	x	x	x
*HLA-DRB1*07:01*	x	x	x	x	x	x	x	x	x	x	x	x	x	x	x	x	x	x	x	x	x	x	x	x	x	x	x	x	x	x	x	x	x	x	x	x	x	x
*HLA-DQA1*05:01*	x	x	x	x	x	x	x	x	x	x	x	x	x	x	x	x	x	x	x	x	x	x	x	x	x	x	x	x	x	x	x	x	x	x	x	x	x	x
*HLA-DQB1*03:01*	x	x	x	x	x	x	x	x	x	x	x	x	x	x	x	x	x	x	x	x	x	x	x	x	x	x	x	x	x	x	x	x	x	x	x	x	x	x
*HLA-DPA1*01:03*	x	x	x	x	x	x	x	x	x	x	x	x	x	x	x	x	x	x	x	x	x	x	x	x	x	x	x	x	x	x	x	x	x	x	x	x	x	x
*HLA-DPB1*04:01*	x	x	x	x	x	x	x	x	x	x	x	x	x	x	x	x	x	x	x	x	x	x	x	x	x	x	x	x	x	x	x	x	x	x	x	x	x	x
*MICA*008:01*	x	x	x	x	x	x	x	x	x	x	x	x	x	x	x	x	x	x	x	x	x	x	x	x	x	x	x	x	x	x	x	x	x	x	x	x	x	x

*: Amino acid sequences corresponding to the most common 4-digit alleles are indicated for the respective HLA genes and *MICA*.

For the definition of HLA gene amino acid positions, the start codon of the mature HLA protein was labeled as position 1, and the codon 5' to this was numbered position -1.

Figure S2. Stepwise regression analysis results of PsA-affected vs control individuals and PsC-affected vs control individuals.

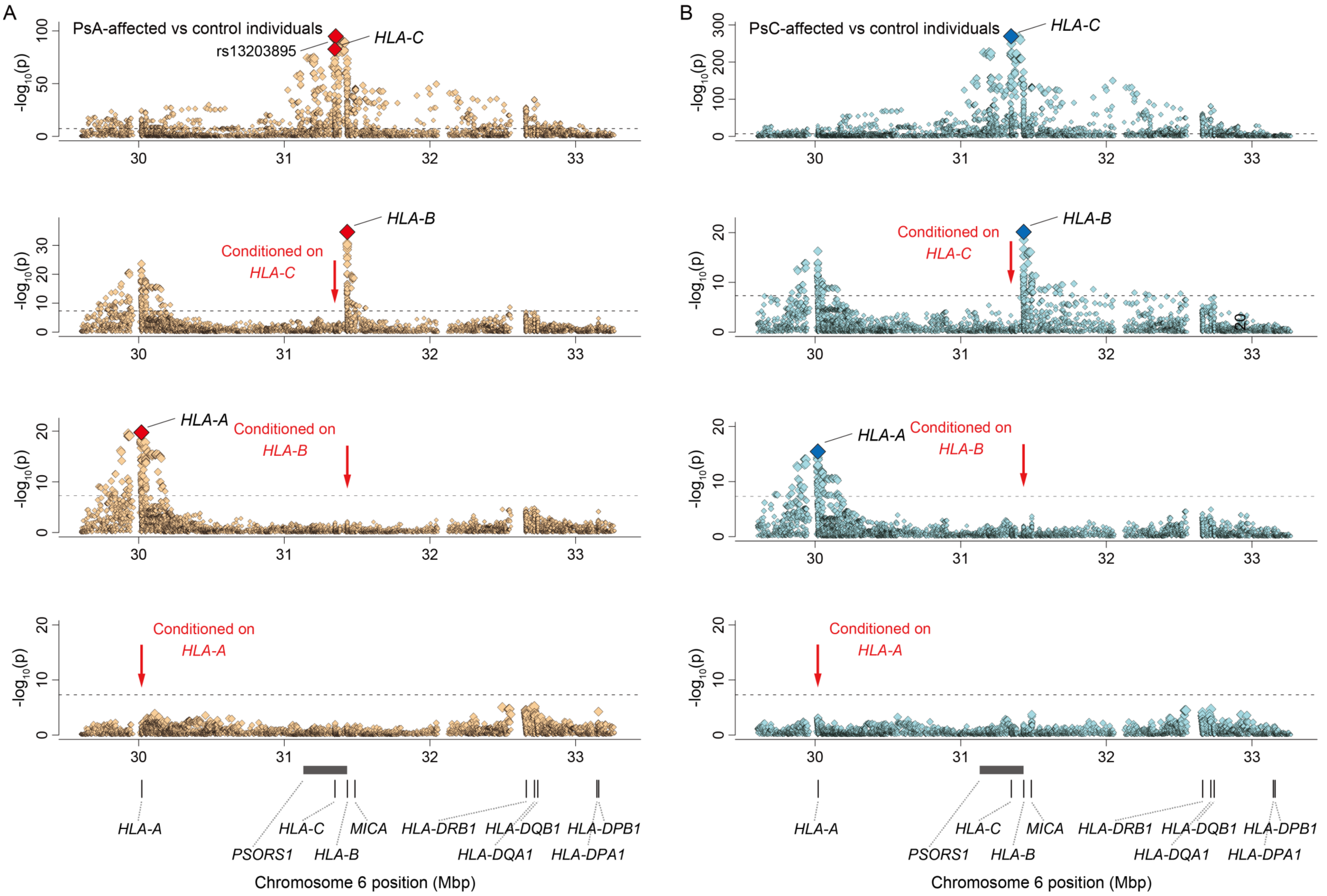

Stepwise conditional associations on (**A**) PsA-affected vs control individuals, (**B**) PsC-affected vs control individuals. Each diamond represents –$\log_{10}(p)$ of the variants, including SNPs, classical HLA or *MICA* alleles, and amino acid polymorphisms of the HLA genes or *MICA*. The dotted horizontal line represents the significance threshold of $P = 5.0 \times 10^{-8}$. The bottom panel shows the physical positions of the HLA genes, *MICA,* and *PSORS1* on chromosome 6 (UCSC Genome Browser hg18).

PsV; psoriasis vulgaris, PsA; psoriatic arthritis, PsC; cutaneous psoriasis.

Figure S3. Three-dimensional ribbon models for the HLA-A and HLA-DQ protein.

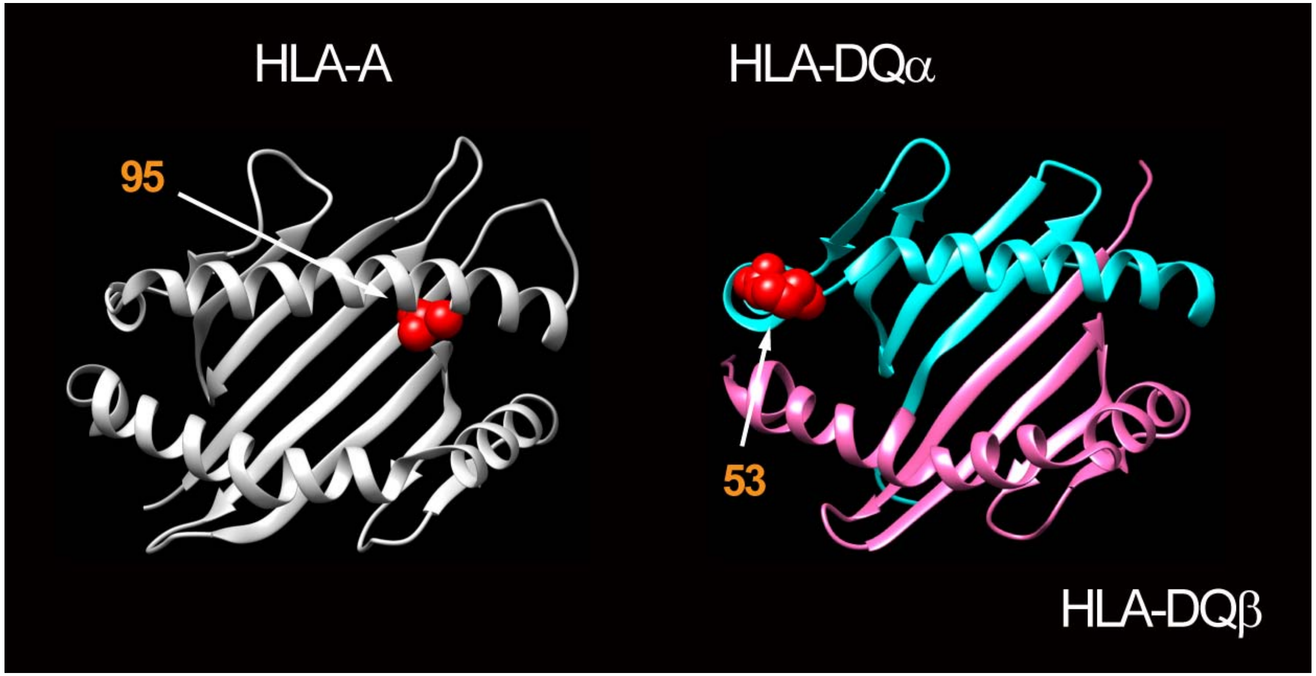

HLA-A and HLA-DQ protein structures are based on Protein Data Bank entries 1x7q and 1jk8, respectively, and prepared using UCSF Chimera version 1.7. Residues at amino acid positions overall PsV risk are highlighted as red spheres.

Figure S4. Linkage disequilibrium among risk HLA variants of class I HLA genes.

r^2 (obtained from European T1DGC reference panel)	HLA-A 95Ile	HLA-A 95Val	HLA-A 95Leu	*HLA-C*06:02*	*HLA-C*12:03*	HLA-B 67Tyr	HLA-B 67Phe	HLA-B 67Ser	HLA-B 67Cys	HLA-B 67Met	HLA-B 45Glu	HLA-B 45Thr	HLA-B 45Lys	HLA-B 45Met	HLA-B 45Gly	HLA-B 9Tyr	HLA-B 9Asp	HLA-B 9His
HLA-A 95Ile	-	-	-	0.00	0.00	0.00	0.02	0.03	0.00	0.00	0.02	0.00	0.02	0.02	0.00	0.01	0.03	0.00
HLA-A 95Val	-	-	-	0.00	0.00	0.00	0.02	0.02	0.00	0.00	0.01	0.01	0.01	0.02	0.00	0.01	0.02	0.00
HLA-A 95Leu	-	-	-	0.00	0.00	0.00	0.00	0.00	0.00	0.00	0.00	0.00	0.00	0.00	0.00	0.00	0.01	0.00
*HLA-C*06:02*	0.00	0.00	0.00	-	-	0.01	0.03	0.03	0.01	0.06	0.05	0.01	0.01	0.08	0.00	0.00	0.01	0.02
*HLA-C*12:03*	0.00	0.00	0.00	-	-	0.00	0.01	0.00	0.07	0.00	0.00	0.01	0.01	0.01	0.00	0.00	0.01	0.00
HLA-B 67Tyr	0.00	0.00	0.00	0.01	0.00	-	-	-	-	-	0.19	0.04	0.04	0.01	0.01	0.08	0.02	0.04
HLA-B 67Phe	0.02	0.02	0.00	0.03	0.01	-	-	-	-	-	0.03	0.11	0.11	0.06	0.00	0.03	0.43	0.12
HLA-B 67Ser	0.03	0.02	0.00	0.03	0.00	-	-	-	-	-	0.43	0.01	0.38	0.08	0.00	0.03	0.13	0.22
HLA-B 67Cys	0.00	0.00	0.00	0.01	0.07	-	-	-	-	-	0.21	0.04	0.04	0.02	0.00	0.00	0.02	0.00
HLA-B 67Met	0.00	0.00	0.00	0.06	0.00	-	-	-	-	-	0.03	0.02	0.01	0.06	0.00	0.03	0.01	0.02
HLA-B 45Glu	0.02	0.01	0.00	0.05	0.00	0.19	0.03	0.43	0.21	0.03	-	-	-	-	-	0.02	0.27	0.07
HLA-B 45Thr	0.00	0.01	0.00	0.01	0.01	0.04	0.11	0.01	0.04	0.02	-	-	-	-	-	0.01	0.06	0.01
HLA-B 45Lys	0.02	0.01	0.00	0.01	0.01	0.04	0.11	0.38	0.04	0.01	-	-	-	-	-	0.04	0.05	0.17
HLA-B 45Met	0.02	0.02	0.00	0.08	0.01	0.01	0.06	0.08	0.02	0.06	-	-	-	-	-	0.10	0.03	0.05
HLA-B 45Gly	0.00	0.00	0.00	0.00	0.00	0.01	0.00	0.00	0.00	0.00	-	-	-	-	-	0.00	0.00	0.00
HLA-B 9Tyr	0.01	0.01	0.00	0.00	0.00	0.08	0.03	0.03	0.00	0.03	0.02	0.01	0.04	0.10	0.00	-	-	-
HLA-B 9Asp	0.03	0.02	0.01	0.01	0.01	0.02	0.43	0.13	0.02	0.01	0.27	0.06	0.05	0.03	0.00	-	-	-
HLA-B 9His	0.00	0.00	0.00	0.02	0.00	0.04	0.12	0.22	0.00	0.02	0.07	0.01	0.17	0.05	0.00	-	-	-

Pairwise linkage disequilibrium matrix (r^2) values among psoriasis risk HLA variants of class I HLA genes, which were calculated based on the haplotype frequencies obtained the from T1DGC European reference panel.

Table S1. Characteristics of the data set collections.

Data set collections	Study design	No. psoriasis-affected individuals				No. control individuals	Total
		PsV	PsV clinical subtypes				
			PsA	PsC	PsA/PsC status unknown		
CASP	GWAS	1,339	340	693	306	1,374	2,713
Genizon		745	137	389	219	964	1,709
Kiel		463	32	269	162	1,130	1,593
PsA		1,414	1,414	0	0	1,385	2,847
CASP-DFU	Targeted follow-up study	1,563	133	575	855	1,141	2,704
PAGE	Immunochip	3,723	982	1,172	1,569	7,595	11,318
Total	-	9,247	3,038	3,098	3,111	13,589	22,884

PsV; psoriasis vulgaris, PsA; psoriatic arthritis, PsC; cutaneous psoriasis.

Table S2. Detailed stepwise association results of the HLA variants with psoriasis risk.

(A Microsoft Excel file for Table S2 is uploaded at the Journal web site.)

Table S3. Association results of the HLA variants on psoriasis risk.

HLA variant	Frequency				PsV-affected vs control individuals		PsA-affected vs control individuals		PsC-affected vs control individuals		PsA-affected vs PsC-affected individuals	
	PsV	PsA	PsC	Control	OR (95%CI)	*P*	OR (95%CI)	*P*	OR (95%CI)	*P*	OR (95%CI)	*P*
Multivaraite full regression model based on PsV-affected vs control individual analysis												
Classical *HLA-C* alleles												
*HLA-C*06:02*	0.23	0.19	0.28	0.093	3.26 (3.02-3.52)	2.1E-201	2.75 (2.43-3.12)	2.4E-56	2.75 (2.43-3.12)	5.9E-139	3.91 (3.52-4.35)	0.043
*HLA-C*12:03*	0.073	0.088	0.065	0.056	1.38 (1.26-1.52)	6.5E-12	1.50 (1.29-1.73)	9.7E-08	1.50 (1.29-1.73)	7.6E-07	1.42 (1.24-1.63)	0.33
Other *HLA-C* 4-digit alleles	0.70	0.72	0.66	0.85	(reference)		(reference)		(reference)		(reference)	
HLA-B amino acid position 67												
Cys	0.16	0.20	0.13	0.12	1.56 (1.45-1.67)	6.0E-35	2.01 (1.80-2.25)	2.8E-35	1.33 (1.19-1.49)	2.7E-07	1.53 (1.28-1.82)	2.0E-06
Met	0.12	0.10	0.14	0.046	1.44 (1.30-1.58)	2.6E-13	1.43 (1.22-1.67)	7.4E-06	1.53 (1.34-1.74)	2.1E-10	0.98 (0.81-1.20)	0.87
Tyr	0.12	0.13	0.11	0.16	1.00 (0.93-1.07)	0.93	1.13 (1.00-1.27)	0.048	0.93 (0.83-1.05)	0.24	1.26 (1.04-1.53)	0.016
Phe	0.21	0.21	0.21	0.26	1.00 (0.93-1.08)	0.99	0.98 (0.86-1.10)	0.70	1.03 (0.92-1.15)	0.60	0.95 (0.78-1.16)	0.63
Ser	0.39	0.36	0.41	0.42	(reference)		(reference)		(reference)		(reference)	
HLA-B amino acid position 9												
Asp	0.096	0.11	0.087	0.11	1.33 (1.21-1.45)	1.6E-09	1.64 (1.41-1.91)	1.0E-10	1.64 (1.41-1.91)	0.035	1.16 (1.01-1.34)	0.0010
Tyr	0.70	0.68	0.72	0.67	(reference)		(reference)		(reference)		(reference)	
His	0.20	0.21	0.19	0.22	0.87 (0.82-0.92)	1.6E-06	0.89 (0.81-0.98)	0.022	0.89 (0.81-0.98)	5.6E-06	0.81 (0.74-0.89)	0.48
HLA-A amino acid position 95												
Val	0.34	0.34	0.34	0.29	1.31 (1.25-1.38)	4.7E-28	1.46 (1.35-1.58)	2.9E-21	1.46 (1.35-1.58)	2.4E-10	1.26 (1.17-1.36)	0.13
Ile	0.56	0.56	0.56	0.59	(reference)		(reference)		(reference)		(reference)	
Leu	0.099	0.098	0.098	0.12	0.89 (0.83-0.95)	7.0E-04	0.94 (0.84-1.06)	0.32	0.94 (0.84-1.06)	0.0011	0.84 (0.75-0.93)	0.61
HLA-DQα1 amino acid position 53												
Arg	0.37	0.36	0.38	0.29	1.07 (1.01-1.13)	0.016	1.10 (1.01-1.20)	0.025	1.10 (1.01-1.20)	0.17	1.06 (0.98-1.14)	0.095
Lys	0.39	0.39	0.38	0.43	(reference)		(reference)		(reference)		(reference)	
Gln	0.25	0.25	0.24	0.29	0.91 (0.86-0.96)	9.3E-04	0.89 (0.81-0.98)	0.013	0.89 (0.81-0.98)	0.097	0.93 (0.85-1.01)	0.89
Association analysis based on PsA-affected vs PsC-affected individual analysis												
HLA-B amino acid position 45												
Glu	0.38	0.43	0.33	0.39	0.90 (0.86-0.94)	4.5E-07	1.14 (1.06-1.22)	1.8E-04	0.76 (0.71-0.81)	1.1E-18	1.46 (1.31-1.62)	2.9E-12
Thr, Lys, Met	0.62	0.57	0.67	0.61	(reference)		(reference)		(reference)		(reference)	

PsV; psoriasis vulgaris, PsA; psoriatic arthritis, PsC; cutaneous psoriasis, OR; odds ratio.

Table S4. Classical 4-digit *HLA-B* alleles corresponding to HLA-B amino acid residues at position 45.

HLA-B amino acid position 45	Classical 4-digit *HLA-B* alleles
Glu	**07:02, *07:05, *08:01, *14:01, *14:02, *15:03, *15:18, *27:02, *27:05, *38:01, *39:01, *39:06, *55:01, *56:01.*
Met	**13:02, *15:01, *15:17, *57:01.*
Lys	**40:01, *40:02, *41:01, *41:02, *44:02, *44:03, *44:05, *45:01, *47:01, *49:01, *50:01.*
Thr	**18:01, *35:01, *35:02, *35:03, *35:08, *37:01, *51:01, *52:01, *53:01, *58:01.*
